# Multifunctional Self-Assembled Block Copolymer/Iron Oxide Nanocomposite Hydrogels Formed from Wormlike Micelles

**DOI:** 10.1021/acsami.4c03007

**Published:** 2024-04-09

**Authors:** Qi Yue, Shiyu Wang, Samuel T. Jones, Lee A. Fielding

**Affiliations:** † Department of Materials, School of Natural Sciences, University of Manchester, Oxford Road, Manchester M13 9PL, U.K.; ‡ Henry Royce Institute, The University of Manchester, Oxford Road, Manchester M13 9PL, U.K.; § School of Chemistry, University of Birmingham, Edgbaston, Birmingham B15 2TT, U.K.

**Keywords:** hydrogels, nanocomposites, nanomaterials, biomaterials, functional materials, polymerization, stimulus-responsive, self-assembly

## Abstract

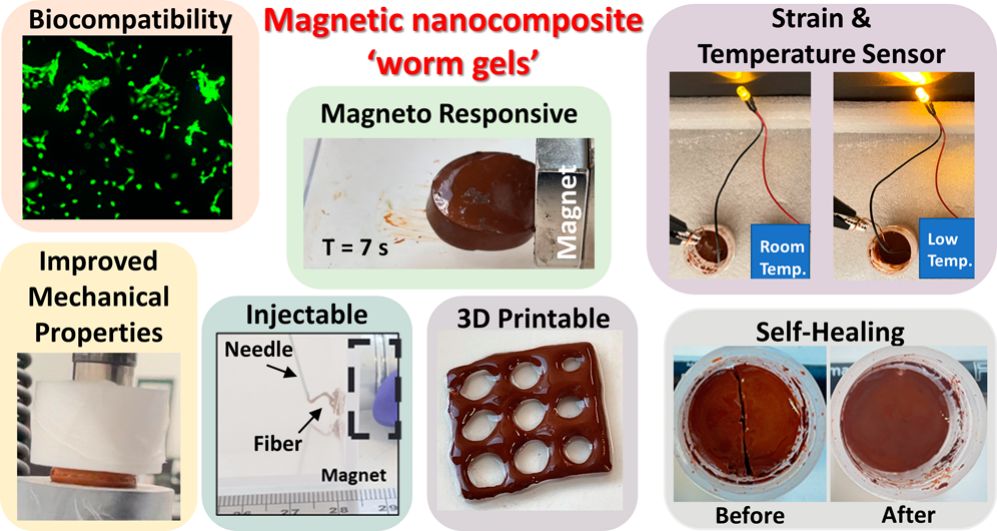

This article reports the preparation of multifunctional magnetic nanocomposite hydrogels formed from wormlike micelles. Specifically, iron oxide nanoparticles were incorporated into a temperature responsive block copolymer, poly(glycerol monomethacrylate)-*b*-poly(2-hydroxypropyl methacrylate) (PGMA-*b*-PHPMA), and graphene oxide (GO) dispersion at a low temperature (∼2 °C) through high-speed mixing and returning the mixture to room temperature, resulting in the formation of nanocomposite gels. The optimal concentrations of iron oxide and GO enhanced the gel strength of the nanocomposite gels, which exhibited a strong magnetic response when a magnetic field was applied. These materials retained the thermoresponsiveness of the PGMA–PHPMA wormlike micelles allowing for a solid-to-liquid transition to occur when the temperature was reduced. The mechanical and rheological properties and performance of the nanocomposite gels were demonstrated to be adjustable, making them suitable for a wide range of potential applications. These nanocomposite worm gels were demonstrated to be relatively adhesive and to act as strain and temperature sensors, with the measured electrical resistance of the nanocomposite gels changing with applied strain and temperature sweeps. The nanocomposite gels were found to recover efficiently after the application of high shear with approximately 100% healing efficiency within seconds. Additionally, these nanocomposite worm gels were injectable, and the addition of GO and iron oxide nanomaterials seemed to have no significant adverse impact on the biocompatibility of the copolymer gels, making them suitable not only for 3D printing in nanocomposite engineering but also for potential utilization in various biomedical applications as an injectable magnetic responsive hydrogel.

## Introduction

Smart soft materials can respond to external environmental stimuli such as temperature, pH, light, electricity, pressure, and magnetism, and are widely used in fields such as soft machines, flexible electronics, shape control, and bioengineering.^1−3^ Among the actuation stimuli mentioned above, magnetic actuation has unique advantages such as the possibility for remote control of materials in confined spaces and high intensity magnetic fields being harmless to organisms.^4,5^ For instance, Sun et al. prepared non-Newtonian, fluid-based, magnetically actuated slime robots, which could negotiate narrow channels.^6^ These robots exhibited various functions such as grasping solid substrates; swallowing and transporting objects; human motion monitoring; and circuit switching and repair. Commonly reported magnetic soft materials include magnetically driven elastomers^7,8^ and magnetic hydrogels,^9,10^ whereby magnetic nanoparticles are typically mixed into a polymer matrix.^11,12^ Magnetic actuating hydrogels are relatively elastic and can be used in microswimming devices, unconstrained small robots, 3D-printed flexible devices, and *in vivo* applications because of their biocompatibility and low toxicity.^8,13−15^ The most common methods reported for the preparation of magnetic hydrogels include physical mixing and *in situ* synthesis.^16^ For *in situ* synthesis, magnetic nanoparticles (MNPs) are either synthesized or incorporated into the hydrogel during the polymerization process.^17^ This method ensures that the magnetic nanoparticles are evenly distributed within the hydrogel matrix, allowing the resulting material to exhibit both the properties of a hydrogel and responsiveness to external magnetic fields.^17^ For example, Mikhnevich et al.^18^ prepared polyacrylamide magnetic hydrogels through free radical polymerization of acrylamide monomer in the presence of magnetic nickel nanoparticles. However, these approaches can be limited by difficulties in nanoparticle dispersion in the polymerization medium and agglomeration occurring during gel formation.^19,20^ In contrast, physical mixing is a relatively simple and straightforward approach to prepare magnetic hydrogels.^21^ In this process, dispersed MNPs become physically trapped within the hydrogel matrix during the gelation process.^22^ For instance, Zhang et al.^23^ reported a superparamagnetic iron oxide nanoparticle (SPION)-loaded nanocapsule hydrogel system for multiple magnetic hyperthermia therapy and long-term magnetic resonance imaging contrast via simply physically mixing poly(organophosphazene) and as-synthesized SPIONs. However, gels formed by physical mixing typically have no strong covalent or chemical bonds between the MNPs and the hydrogel network. Instead, the MNPs are held in place by the physical entanglement of the hydrogel polymer chains, van der Waals forces, and other intermolecular interactions.^16^ Therefore, MNPs may weakly interact with the gel matrix and be separated from it under the influence of external conditions such as strong magnetic fields.^24^

An alternative approach to form polymeric hydrogels is through the use of self-assembled wormlike micelles. A widely reported approach to form these so-called “worm gels” at relatively high copolymer concentrations is via reversible addition–fragmentation chain-transfer (RAFT) mediated polymerization-induced self-assembly (PISA).^25−29^ Thus, a potential route to improve upon the properties of previously reported magnetic gels prepared by physical mixing is to utilize block copolymer self-assembled wormlike micelles as the gel matrix, as opposed to molecularly entangled polymer chains.

In previous reports from our group, poly(glycerol monomethacrylate)-*b*-poly(2-hydroxypropyl methacrylate) (PGMA-*b*-PHPMA) copolymer worm gels with drastically improved mechanical properties and self-healing capabilities were prepared through the incorporation of graphene oxide (GO) into the gel matrix. This was achieved either by physical mixing,^30^ whereby GO was mixed with preformed copolymer at low temperature, or by *in situ* polymerization,^31^ where PGMA was block extended with HPMA in the presence of GO. The latter strategy resulted in the more significant improvements in gel properties, potentially due to chain grafting of polymer to GO occurring during *in situ* radical polymerization resulting in covalent bonding between the two materials.^32,33^ In addition, at room temperature, the PGMA–*b*-PHPMA worm gels exhibit a gel-to-liquid transition upon cooling to ∼5 °C.^27,34−38^ This reversible degelation transition is associated with a loss of worm entanglement which occurs due to a worm-to-sphere order–order transition on cooling.^38−40^ This thermoresponsive behavior, and the shear-thinning properties of the non-GO-containing worm gels, is retained on the inclusion of GO into these copolymer worm gels,^30,31^ and the combined properties allows them to be readily 3D-printed.^31^

**Figure 1:**
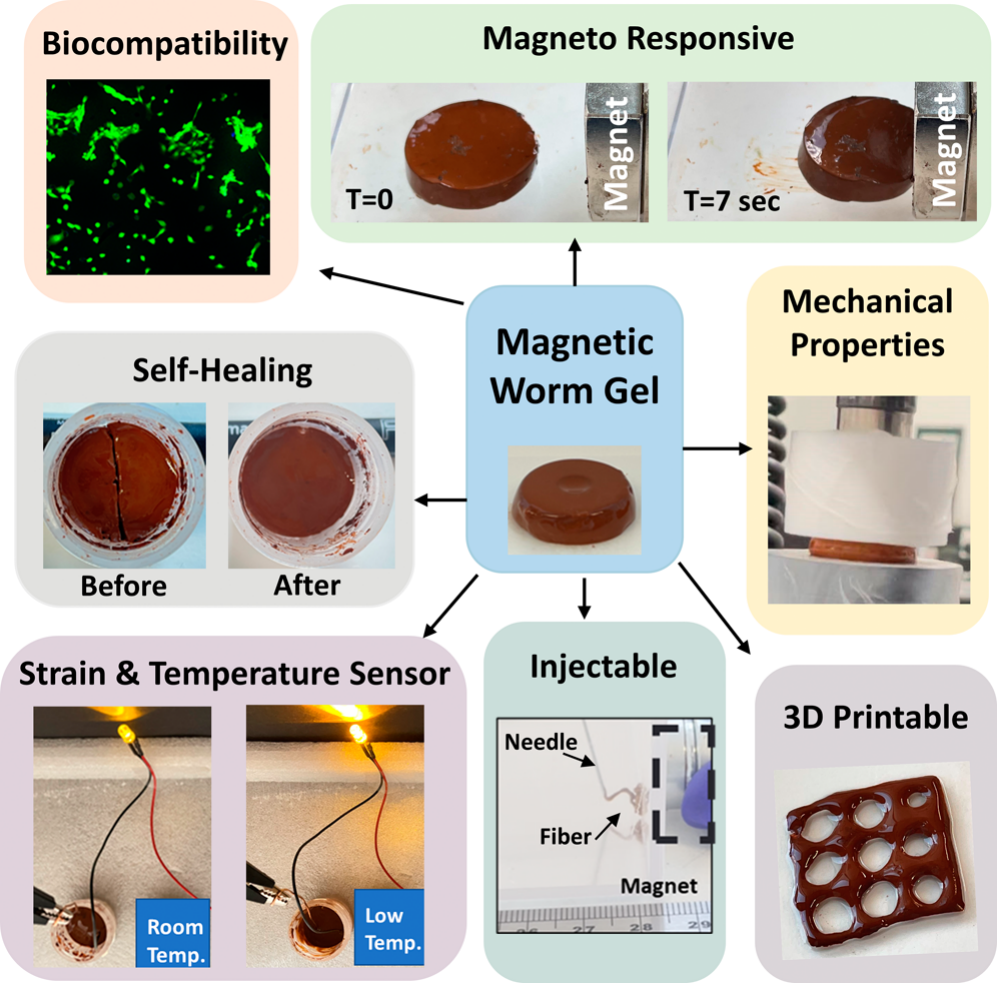
Summary of the multifunctional properties and potential applications of iron oxide-containing PGMA-*b*-PHPMA copolymer nanocomposite worm gels.

Herein, this approach is extended to demonstrate that the incorporation of magnetic iron oxide nanoparticles (IOPs) into PGMA-*b*-PHPMA block copolymer worm gels facilitates the formation of magnetic hydrogels with a very wide range of functional properties. Specifically, PGMA_62_-*b*-PHPMA_170_ (G_62_–H_170_) worm gels prepared by RAFT-mediated dispersion polymerization were employed for the preparation of magnetic nanocomposite copolymer worm gels. IOP powders were rapidly mixed with the copolymer at low temperature, and upon returning to room temperature, uniform nanocomposite worm gels were successfully obtained. In addition, based on our knowledge and expertise that GO can further improve upon the properties of such gels and provide access to a wider performance parameter range, ternary nanocomposite gels containing G_62_–H_170_, IOPs, and GO were investigated. The mechanical and recovery properties of the prepared nanocomposite gels were characterized via oscillatory rheology and tensile testing. Furthermore, for optimized iron oxide loadings, the functional properties of these nanocomposite gels were demonstrated in terms of their temperature and strain sensing abilities, 3D printability, temperature-responsive behavior, adhesive properties, self-healing ability, magnetic response, and biocompatibility (Figure 1).

## Experimental Section

### Materials

All reagents were purchased from Sigma-Aldrich (U.K.) and used as received, unless otherwise noted. Iron(III) oxide nanoparticle powder (20–40 nm average particle size) was purchased from Alfa Aesar and used as received. G_62_–H_170_ and G_62_–H_170_–4% GO were prepared in-house following previously published protocols, which are described in the Supporting Information.^30,31^ Dulbecco’s modified Eagle’s medium (DMEM) was purchased from Thermo Fisher Scientific (U.K.). Fetal bovine serum (FBS) was purchased from Gibco (U.K.), and live/dead assays were purchased from Life Technologies (U.K.). Deionized water was used in all of the experiments.

### Preparation of Fe_2_O_3_-Containing Nanocomposite Worm Gels

*x*% Fe_2_O_3_ G_62_–H_170_ and *x*% Fe_2_O_3_ G_62_–H_170_–4% GO nanocomposite worm gels were prepared following the same protocol, where *x* represents the % w/w of Fe_2_O_3_ based on the copolymer. In all cases, the total copolymer concentration was fixed at 20% w/w, unless otherwise noted. As an example for 20% Fe_2_O_3_ G_62_–H_170_–4%GO, 2 g of G_62_–H_170_–4% GO nanocomposite worm gel and 0.67 g of NaCl solution (1 M) were added into a high-speed mixer container at ∼2 °C. 0.53 g of Fe_2_O_3_ powder was added to the container and allowed to equilibrate at ∼2 °C for 15 min. The mixture was then slowly stirred by hand with a spatula before the container was sealed and placed in a high-speed mixer (Hauschild, DAC 150SP). The high-speed mixer was run at 1000 rpm for 1 min, and then the sample container was removed and placed in an ice box held at approximately 2 °C for 1 min. The container was then mixed again at 500 rpm for 30 s before being allowed to return to room temperature for analysis.

### Mechanical Property Testing

The mechanical performance of Fe_2_O_3_-containing nanocomposite worm gels was determined by using an Instron 5564H1580 universal testing machine equipped with a 10 N load cell. Gels were prepared for tensile testing by initially casting the gels in a rectangular mold (3.7 × 1.0 cm, thickness 0.2 cm) placed on a PTFE-coated glass slide and attached to a paper frame prior to testing. When mounted, the paper frame was cut and strain was applied to the samples at a rate of 8 mm min^–1^. Young’s moduli were calculated from the gradient of the obtained tensile stress–strain curves in the initial linear region. Toughness was calculated by integrating the area under the stress–strain curves. Self-healing tests were performed by placing the broken gels after initial tensile testing back into a rectangular mold, which was then firmly closed and sealed for 4 h at room temperature. After the self-healing process, tensile testing was conducted using the same method as described above. Self-healing efficiency was calculated by dividing the measured toughness of the healed samples by the toughness of the original samples. Each measurement described above was conducted in triplicate. For uniaxial compression tests, the nanocomposite gels were prepared in cylindrical plastic molds with dimensions of 11.5 mm height and 11.0 mm diameter. Gels were compressed between two plates at a strain rate of 1 mm min^–1^ until either the maximum load value was reached or fracture was observed. Adhesive strength was characterized by lap shear testing. Substrates (75 × 25 × 1 mm) including glass, plastic, wood, and metal were cleaned via sonication in water and ethanol prior to testing. Nanocomposite gels were sandwiched between the two substrates 15 s prior to each measurement, and shear was applied at a rate of 2 mm min^–1^.

### 3D Printing of Gels

Fe_2_O_3_-containing nanocomposite worm gels were 3D-printed using a robot printer (I&J7300R-LF, Fisnar Inc., WI, USA). Samples were loaded into a 5 mL pressure-driven syringe at room temperature and pneumatically printed through a nozzle (diameter = 840 μm, length = 1.27 cm) with a head speed of 5 mm s^–1^. Thin-walled letters were printed onto a stainless steel substrate, and QR codes (12.5 × 12.5 cm) were 3D-printed using 3 layers onto cardboard. The smallest printed square in each QR code was 5 × 5 mm.

### Rheology Measurements

A MARS iQ Air rheometer (HAAKE Instruments) equipped with a variable-temperature Peltier plate and a 40 mm 2° titanium cone was used for all experiments. An oscillatory mode was used to measure storage modulus (*G*′) and loss modulus (*G*″) as a function of percentage strain. Percentage strain amplitude sweeps were conducted between 0.01 and 130 rad s^–1^ at a constant temperature of 25 °C, with a frequency of 10 rad s^–1^. For shear thinning recovery experiments, samples were tested in time sweeps by alternating cycles of recovery at low shear (5 min, 0.2% strain) and high shear (5 min, 100% strain) at 10 rad s^–1^. Recovery efficiency was calculated from the ratio of final *G*′ measurement to the initial value of *G*′. Percentage strain amplitude as a function of temperature was used to assess the critical gelation temperature (CGT) and gel strength. Temperature sweeps were conducted using an applied strain amplitude of 1.0% at an angular frequency of 10 rad s^–1^. The temperature was reduced from 25 to 2 °C in 1 °C intervals, allowing for 3 min of thermal equilibrium at each step. After 5 min at 2 °C, the sample was heated back to 25 °C in 1 °C intervals. For multiple cooling–heating cycle experiments, *G*′ was measured at 25 and 2 °C alternately with an applied strain amplitude of 1.0% at an angular frequency of 10 rad s^–1^.

### Electrical Measurements

All electrical measurements were performed using a Keysight 34465A 6.5 digital multimeter. For compression sensing and temperature sensing experiments, the nanocomposite worm gels were modeled in a cylindrical container (diameter = 1.25 cm and thickness = 0.5 cm). Electrical resistance was measured in real time as a function of either applied compression or temperature change. Copper wires were connected to the top and bottom of the sample secured with carbon tape, and connected to the multimeter and a light-emitting diode (LED). During compression and temperature variation, resistance was recorded in intervals of 1 and 5 s, respectively. For stretching experiments, the gels were molded into rectangles (3.7 × 1.0 cm, thickness 0.2 cm), and copper wires from the multimeter were attached to two metallic spatulas self-adhered to each side of the gel. Resistance was recorded in 10 s intervals as the gel was stretched by increasing the distance between the two spatulas.

### Cell Culture

The 3T3 cell line (mouse embryonic fibroblasts) was used in the following live/dead and MTS assays. Cells were cultured in Dulbecco’s modified Eagle’s medium (DMEM) supplemented with 10% fetal bovine serum and antibiotic/antimycotic at 37 °C in a humidified 5% CO_2_ atmosphere.

### Gel Sterilization and Preparation

Prior to cell seeding, all gels within the well plates were sterilized under UV light for 1 h in a biosafety cabinet. Throughout the exposure, the plate was covered with a lid and water was added to the remaining empty wells to maintain a moist environment and prevent the gels from drying out. Subsequently, the gels were submerged in DMEM overnight. The medium was then removed before the seeding process.

### Live/Dead Assay

Three groups of gels (40 mg × 4 samples) were placed onto 13 mm glass coverslips in 24 well plates, cooled in the fridge, and allowed to form ∼6 mm gel disks. After gel sterilization and preparation (described above), 3T3 cells were seeded at a density of 1 × 10^5^ per well and cultured for 8 h. Media were changed daily after this point. At the time points of 24, 48, and 72 h since seeding, a group containing 4 samples and 1 NTC (no template control, where cells were seeded in wells without gel samples) was treated with live/dead reagents as per the manufacturer’s instructions to observe the cellular activity. Discarding the old media, 200 μL PBS solutions containing reagents were added to each well, and samples were incubated for 20 min in a biosafety cabinet. A foil cover was placed on the plate to shield it from light. After delicately rinsing with PBS buffer, each coverslip was carefully transferred onto a mounting solution droplet on a glass slide by fine-tipped forceps, with the gel side facing upward. Throughout the transfer, the forceps made only minimal contact with the edge of the coverslip, ensuring minimal disruption to the cell coating. Subsequent imaging was performed by using a Leica SP8 confocal fluorescence microscope.

### MTS Assay

Three groups of gels (20 mg × 4 samples × 3 duplicates) were placed into 96-well plates, cooled in the fridge, and allowed to partially cover the bottoms of the wells. After gel sterilization and preparation (described above), 3T3 cells were seeded at a density of 2.5 × 10^4^ per well and cultured for 8 h. Media were changed daily after this point. After 1, 4, and 7 days since cell seeding, a group containing 4 samples and 1 NTC was treated with MTS reagents to measure cellular metabolic activity. The cells were washed with PBS buffer, and then 120 μL of fresh DMEM media and 30 μL of MTS reagents were added to each well. Samples were incubated for 4 h in a humidified, 5% CO_2_ atmosphere and, following the incubation period, 100 μL of the media was collected and subjected to a brief centrifugation cycle. The absorbance of the resulting solution was measured at 490 nm by using an EnVision Nexus Multimode Microplate Reader.

## Results and Discussion

### Preparation of *x*% Fe_2_O_3_ PGMA-*b*-PHPMA and *x*% Fe_2_O_3_ PGMA-*b*-PHPMA–4% GO Nanocomposite Worm Gels

The synthesis of PGMA-*b*-PHPMA block copolymer worm gels by RAFT-mediated PISA has been widely reported.^30,35,36,41,42^ G_62_–H_170_ was synthesized by block extending a PGMA_62_ macromolecular chain-transfer agent (Figure S1a) with HPMA via RAFT aqueous dispersion polymerization at 20% w/w copolymer concentration. Similarly, G_62_–H_170_–4% GO nanocomposite gels were prepared by *in situ* polymerization by chain extending a PGMA_62_ macromolecular chain-transfer agent with HPMA via RAFT aqueous dispersion polymerization at 20% w/w copolymer concentration in the presence of 4% w/w GO based on copolymer (Figure S1b).^31^ In both cases, the target degree of polymerization of the core-forming PHPMA block was fixed at 170 to obtain free-standing gels.^43^

**Figure 2:**
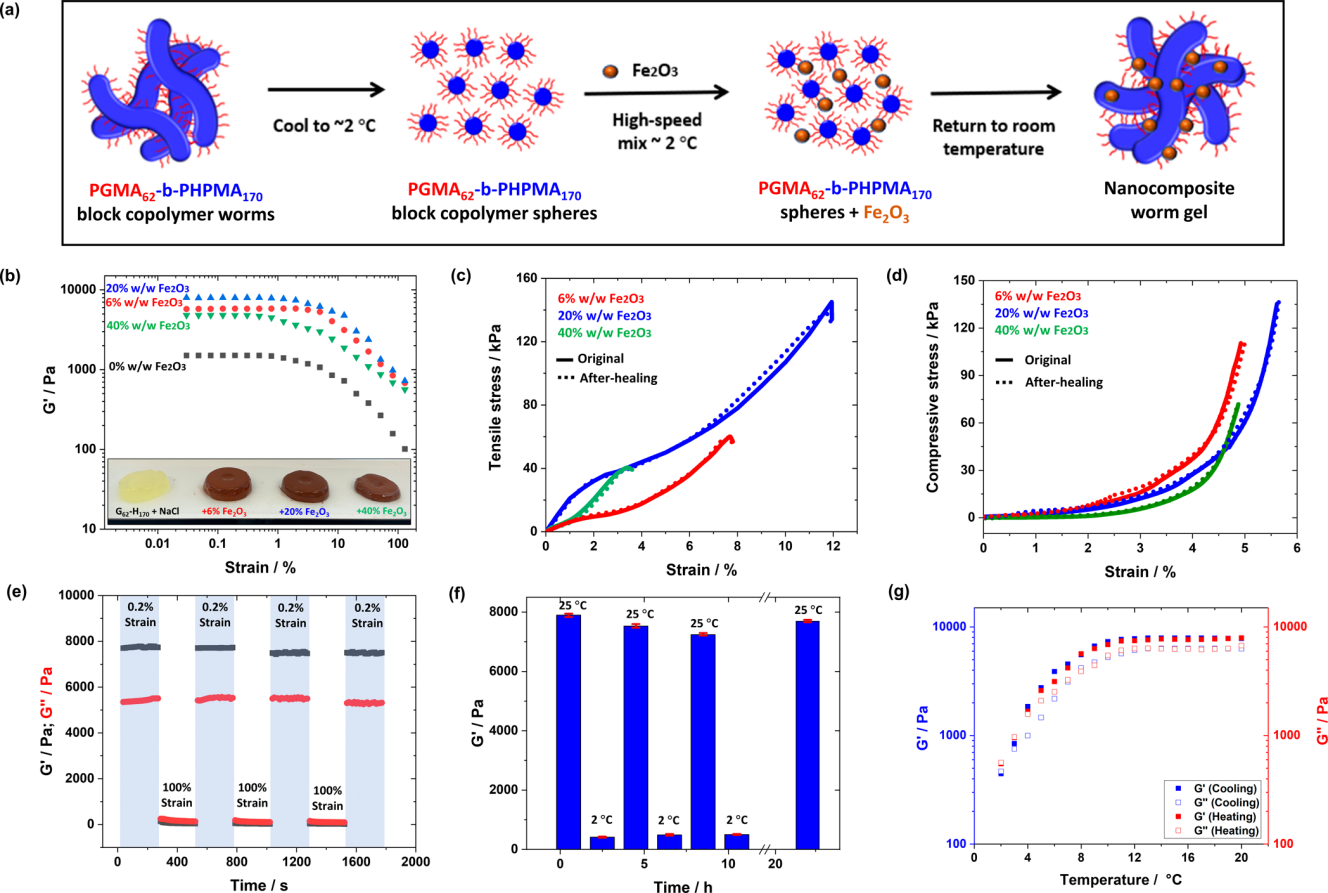
(a) Schematic representation of the preparation of *x*% Fe_2_O_3_ G_62_–H_170_ nanocomposite worm gels. (b) Storage modulus (*G*′) versus % strain for *x*% Fe_2_O_3_ G_62_–H_170_ nanocomposite worm gels (*x* = 0, 6, 20, 40); inset: photographs of gels studied. (c) Tensile testing data for *x*% Fe_2_O_3_ G_62_–H_170_ nanocomposite worm gels at room temperature. Solid lines represent the initial gel, and dashed lines are after allowing the fractured gels to self-heal. (d) Compression stress–strain curves for *x*% Fe_2_O_3_ G_62_–H_170_ nanocomposite worm gels at room temperature (solid lines: original and dashed lines: after self-healing). (e) Oscillatory rheology data for a 20% Fe_2_O_3_ G_62_–H_170_ nanocomposite worm gel measured using a continuous strain sweep with alternating strain (γ = 0.2 and 100%) at 25 °C with an angular frequency of 10 rad s^–1^. (f) Temperature-dependent storage modulus determined by oscillatory rheology for a 20% Fe_2_O_3_ G_62_–H_170_ nanocomposite worm gel. The temperature was varied from 25 to 2 to 25 °C with 2 h equilibration at each step. The final step was a 12 h equilibration at 25 °C. All measurements were conducted at an angular frequency of 10 rad s^–1^ and an applied strain amplitude of 1.0%. (g) Temperature-dependent oscillatory rheology of a 20% Fe_2_O_3_ G_62_–H_170_ nanocomposite worm gel. The temperature was varied from 20 to 2 °C to 20 °C in 1 °C steps with 3 min equilibration at each step. All measurements were conducted at an angular frequency of 10 rad s^–1^ and applied strain amplitude of 1.0%.

**Figure 3:**
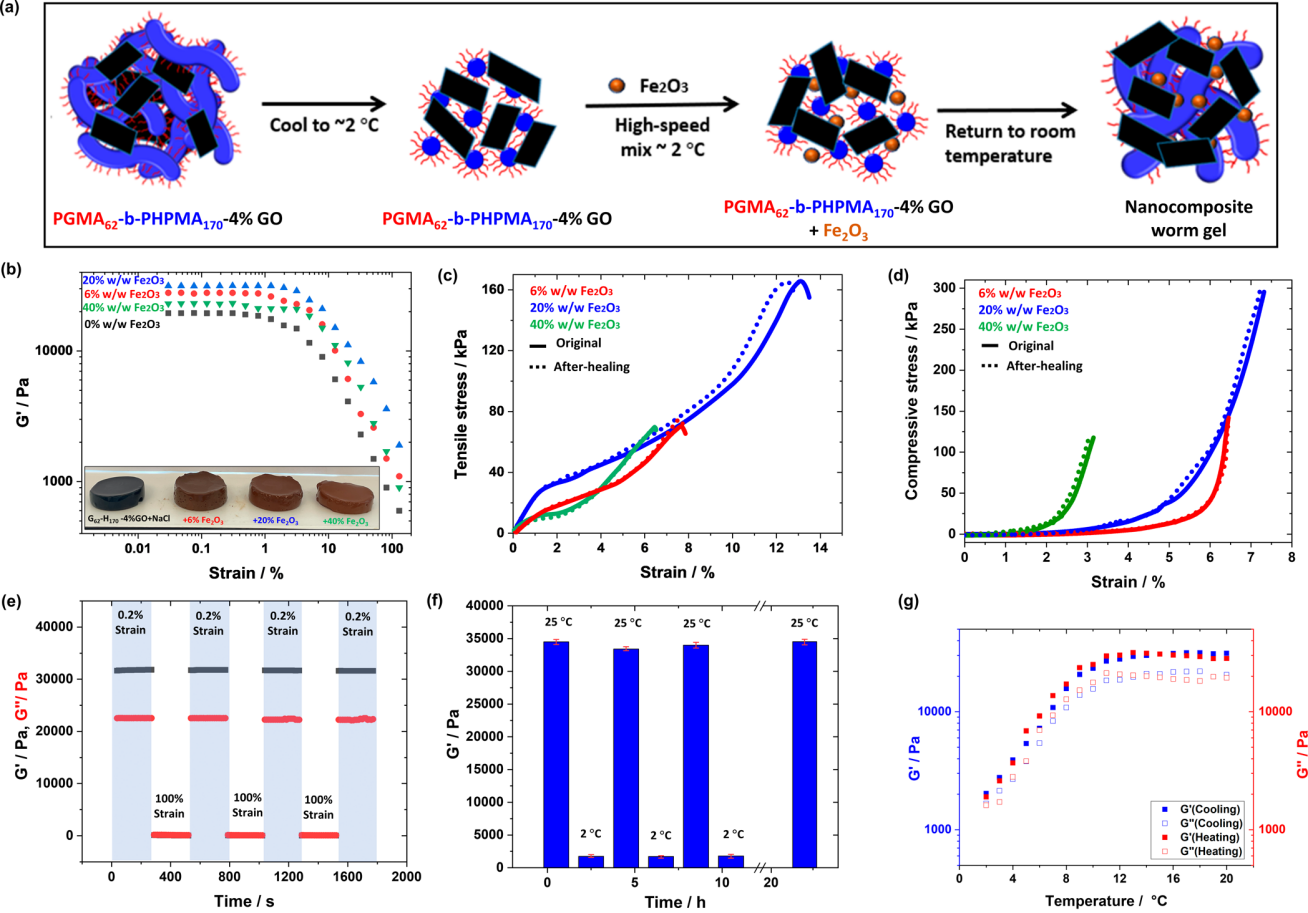
(a) Schematic representation of the preparation of *x*% Fe_2_O_3_ G_62_–H_170_–4% GO nanocomposite worm gels. (b) Storage modulus (*G*′) versus % strain for *x*% Fe_2_O_3_ G_62_–H_170_–4% GO nanocomposite worm gels (*x* = 0, 6, 20, 40); inset: photographs of gels studied. (c) Tensile testing data for *x*% Fe_2_O_3_ G_62_–H_170_–4% GO nanocomposite worm gels (solid lines: initial gels, and dashed lines: after self-healing) at room temperature. (d) Compression stress–strain curves for *x*% Fe_2_O_3_ G_62_–H_170_–4% GO nanocomposite worm gels at room temperature (solid lines: initial gels, and dashed lines: after self-healing). (e) Oscillatory rheology data for a 20% Fe_2_O_3_ G_62_–H_170_–4% GO nanocomposite worm gel measured using a continuous strain sweep with alternating strain (γ = 0.2 and 100%) at 25 °C with an angular frequency of 10 rad s^–1^. (f) Temperature-dependent storage modulus obtained by oscillatory rheology for a 20% Fe_2_O_3_ G_62_–H_170_–4% GO nanocomposite worm gel. The temperature was varied from 25 to 2 to 25 °C s with 2 h equilibration at each step. The final step was a 12 h equilibration at 25 °C. All measurements were conducted at an angular frequency of 10 rad s^–1^ and an applied strain amplitude of 1.0%. (g) Temperature-dependent oscillatory rheology for a 20% Fe_2_O_3_ G_62_–H_170_–4% GO nanocomposite gel. The temperature was varied from 20 to 2 °C to 20 °C in 1 °C steps with 3 min equilibration at each step. All measurements were conducted at an angular frequency of 10 rad s^–1^ and applied strain amplitude of 1.0%.

*x*% Fe_2_O_3_ nanocomposite worm gels were prepared using a high-speed mixer after adding iron oxide nanoparticles to the copolymer dispersions at low temperature, exploiting the reversible degelation transition which these copolymer gels undergo on cooling due to a worm-to-sphere morphological transition (Figures 2a and 3a).^29,36,44^

γ-Fe_2_O_3_ soft magnetic nanoparticles from a commercial source were chosen for use in this investigation as they have lower coercivity, higher electrical resistivity, and good thermal stability when compared to other kinds of IOPs.^45−48^ In addition, we recently reported the preparation of high solids dispersions of these IOPs for 3D printing using the same high-speed mixer and PGMA as a polymeric additive.^48^ In this prior study, the mean diameter of these IOPs was determined to be 29 ± 9 nm by SEM. However, DLS studies of 0.1% w/w IOP dispersions indicated that some aggregates remained after dispersion of the IOP power in water.^48^ Additionally, TEM images obtained for pristine IOPs also suggest the presence of aggregates on dispersion into water at high dilutions (Figure S4). Nevertheless, after sufficient high-speed mixing with copolymer at low temperature, the Fe_2_O_3_/G_62_–H_170_ dispersions formed a stable viscous fluid, and the IOPs did not separate from the dispersion under the application of a strong magnetic force. Given that PGMA is a nonionic and water-soluble polymer which has pendant 1,2-diol functional groups, it is anticipated that this polymer has the capability to adsorb onto the surface of IOPs through hydrogen bonding and the formation of a five-membered chelate ring with Fe,^48,49^ thus helping to disperse the IOPs during mixing.

After subsequent reheating to room temperature, free-standing nanocomposite gels were reformed with Fe_2_O_3_ loadings between 6 and 40% w/w, based on copolymer (Figures 2b and 3b, insets). Our previous work demonstrated that regardless of whether “physical mixing” or “*in situ* polymerization” was used to prepare nanocomposite worm gels, the introduction of GO only resulted in reinforced worm gel formations within relatively narrow concentration ranges (<6% w/w GO, based on copolymer).^30,31^ In addition, relatively high concentrations of incorporated GO prevented sphere-to-sphere fusion to (re)form worms, resulting in low-viscosity dispersions being formed after mixing. Therefore, a high-speed mixer was used in this study. This had the combined effect of reducing the time taken to formulate these gels and improving the properties of the prepared nanocomposite gels, when compared to simply preparing the mixtures using a magnetic stirrer (Figures S5a and S6). Furthermore, up to 40% w/w Fe_2_O_3_ based on copolymer could be incorporated into both G_62_–H_170_ and G_62_–H_170_–4% GO dispersions, with gel reformation occurring in all cases upon returning to room temperature. TEM images (Figure S7) confirmed that after high-speed mixing at low temperature and returning to room temperature, copolymer worms were present and the Fe_2_O_3_ nanoparticles were uniformly distributed throughout the mixture.

### Physical Properties of *x*% Fe_2_O_3_ PGMA-*b*-PHPMA Nanocomposite Worm Gels

The mechanical properties of the nanocomposite worm gels were investigated using oscillatory rheology and tensile and compression testing (summarized in Tables S1 and S2). The measured gel strengths (*G*′) varied as the Fe_2_O_3_ loading was increased from 0 to 40% w/w, based on copolymer. For *x*% Fe_2_O_3_ G_62_–H_170_ gels, the measured gel strength increased from 1.5 to 7.9 kPa as the Fe_2_O_3_ concentration increased from 0 to 20% w/w, based on copolymer and on increasing the Fe_2_O_3_ content to 40% w/w, the measured gel strength reduced to 4.8 kPa (Figures 2b and S6a). Thus, as we have reported for GO-containing gels,^30,31^ there is clearly an upper limit to the volume fraction of IOPs within these gels which provides improved mechanical properties but the rationale as to what concentration this corresponds to is not yet fully understood.

The shear-thinning and recovery properties of these nanocomposite gels were investigated by oscillatory rheology experiments whereby the shear strain was varied between 0.2% and 100% in 5 min intervals. The 20% w/w Fe_2_O_3_ G_62_–H_170_ gel, which exhibited the maximum gel strength, also had the highest healing efficiency of approximately 95% (Figure 2e). In all cases, when the lower initial shear strain (0.2%) was applied, the nanocomposite gels showed solid-like behavior (*G*′ > *G*″) within the linear viscoelastic region. When the higher shear strain (100%) was applied, the nanocomposite worm gels tended to a liquid-like state (*G*″ > *G*′) and shear thinned. After removal of the high shear strain, the samples recovered almost immediately. After 7 cycles, the recovery efficiency did not reduce further for all samples, with healing efficiencies between 81% and 95% (Figure S8a).

Temperature response behavior was investigated by variable-temperature rheology studies (Figures 2g and S9, top row). The critical gelation temperature (CGT) of *x*% Fe_2_O_3_ G_62_–H_170_ nanocomposite worm gels on cooling was between 2 and 9 °C and from 3 to 8 °C on heating (Table S1). The reversible degelation behavior over seven heating–cooling cycles (2 h for each step) was investigated (Figure 2f) and, similar to that of the shear-thinning recovery tests in Figure 2e, the *x*% Fe_2_O_3_ G_62_–H_170_ nanocomposite worm gels showed highly efficient reversible gelation behavior. *G*′ for the 20% Fe_2_O_3_ G_62_–H_170_ nanocomposite worm gel gradually reduced from 7.9 to 7.3 kPa after 10 h of alternating temperature cycles and, after being returned to room temperature for 12 h, the value of *G*′ recovered but was slightly lower than the initially measured value (Figure 2f).

The *x*% Fe_2_O_3_ G_62_–H_170_ nanocomposite worm gels also showed excellent recovery performance when subjected to compression (Figures 2d and S8b and Table S2). The compressive modulus increased from 3.9 to 4.5 kPa as the Fe_2_O_3_ concentration was increased from 6% to 20% and then reduced to ∼2.0 kPa when increased to 40%. Additionally, the fractured samples were remolded for 4 h at room temperature (Figure S10) and tested again. The stress–strain curves of the self-healed *x*% Fe_2_O_3_ G_62_–H_170_ composite worm gels overlapped almost completely with the original composite worm gels indicating rapid self-healing behavior for each case (Figure 2d). For instance, a stress of ∼135 kPa at ∼6% strain was recorded before fracture for the 20% Fe_2_O_3_ G_62_–H_170_ nanocomposite worm gel with a compressive modulus of ∼4.5 kPa. After self-healing, the stress and strain were similar to the original composite worm gel (∼140 kPa, 6.2% strain, and compressive modulus of ∼4.7 kPa).

Tensile testing data for *x*% Fe_2_O_3_ G_62_–H_170_ nanocomposite worm gels as prepared and after healing are shown in Figure 2c and summarized in Table S2. The stress–strain curves had an initial linear gradient up to ∼0.8% strain before yielding. After strain hardening, these gels were extended and then fractured. The 20% Fe_2_O_3_ nanocomposite worm gels displayed the best tensile performance with a Young’s modulus of ∼18 kPa and fracture strain of ∼12% (Figure S8c,d). The fractured samples were recast in molds and allowed to heal at room temperature for 4 h before retesting. The Young’s modulus for 20% Fe_2_O_3_ G_62_–H_170_ was ∼20 kPa, with a fracture strain of ∼11.7 kPa, which was similar to the original composite gel. It is worth noting that for both compression and tensile tests, the curves after self-healing (dotted lines) overlapped almost completely with the original curves, further indicating the excellent self-healing properties of these gels. As expected, an increase in the Fe_2_O_3_ nanoparticle concentration improved the mechanical performance up to a certain point, upon which a decrease in properties was observed. These observations followed the same trends observed in oscillatory rheology studies (Figure 2b).

### Physical Properties of *x*% Fe_2_O_3_ PGMA-*b*-PHPMA–4% GO Nanocomposite Worm Gels

In previous work, the addition of GO to copolymer worm gels (in the absence of iron oxide nanoparticles) was demonstrated to improve their mechanical properties and functional behavior.^30,31^ PGMA-*b*-PHPMA gels containing 4% w/w GO, based on copolymer, prepared by *in situ* RAFT aqueous dispersion polymerization of HPMA in the presence of GO were found to have optimal mechanical and self-healing properties. It was therefore hypothesized that the inclusion of GO at this concentration into the Fe_2_O_3_-containing worm gels reported herein would further improve their performance. As expected, tensile, compression, and rheological studies indicated that the GO-containing IOP/worm gels displayed improved properties and self-healing behavior compared with nanocomposite gels without GO (Tables S1 and S2). The gel strength (*G*′) for *x*% Fe_2_O_3_ G_62_–H_170_–4% GO nanocomposite gels increased from 19.4 to 31.5 kPa as the Fe_2_O_3_ concentration increased from 0% to 20% based on copolymer (Figure 3b). However, *G*′ decreased to ∼23.0 kPa as the Fe_2_O_3_ concentration increased to 40% w/w. While these values of *G*′ were 4 to 5 times higher than for gels without GO present, the trend observed for variation in *G*′ with Fe_2_O_3_ concentration was similar. Furthermore, it was also observed that the composite worm gels from both series exhibited the best performance when 20% w/w Fe_2_O_3_, based on the copolymer, was used. The shear-thinning and recovery behavior of the *x*% Fe_2_O_3_ G_62_–H_170_–4% GO nanocomposite gels was investigated under the same conditions as the *x*% Fe_2_O_3_ G_62_–H_170_ nanocomposite gels (Figure 3e). The GO-containing gels not only recovered immediately to a solidlike state but also showed an improved healing efficiency (∼90% to ∼99%, Figure S11a) over the gels without GO.

Variable-temperature rheology studies were conducted on the GO- and Fe_2_O_3_-containing composite gels between 2 and 20 °C using an applied strain of 1.0%. All samples displayed a decrease in *G*′ as the temperature reduced to 2 °C (Figures 3g and S9, bottom row). However, only the 40% Fe_2_O_3_ G_62_–H_170_–4% GO sample exhibited a crossover of *G*′ and *G*″ on both cooling (6 °C) and heating (7 °C). For Fe_2_O_3_ concentrations of 6% and 20%, although the *G*′ and *G*″ values were lowered significantly, no CGT could be determined, except for 6% Fe_2_O_3_ G_62_–H_170_–4% GO on heating (3 °C) (Table S1). Thus, although the rheological properties of the *x*% Fe_2_O_3_ G_62_–H_170_–4% GO are increased compared to *x*% Fe_2_O_3_ G_62_–H_170_ gels, the GO-containing gels lose some of their rapid temperature-responsive behavior. Reversible degelation over several heating–cooling cycles was investigated (Figure 3f). For the 20% Fe_2_O_3_ G_62_–H_170_–4% GO sample, reversible behavior was observed over seven heating–cooling cycles (2 h for each step) and *G*′ remained almost equal to the initial *G*′ value of ∼34 kPa. After being held at room temperature for 12 h, *G*′ remained constant at 34.5 kPa.

The *x*% Fe_2_O_3_ G_62_–H_170_–4% GO nanocomposite worm gels exhibited better compressive properties than those without GO (Table S2). For 20% Fe_2_O_3_ G_62_–H_170_–4% GO, the compression stress was ∼295 kPa and no fracture occurred at 7.3% strain, even after being repeatedly compressed (Figure 3d). For the *x*% Fe_2_O_3_ G_62_–H_170_–4% GO worm gels with Fe_2_O_3_ concentrations of 6% w/w and 40% w/w the compression stress was lower than for 20% w/w Fe_2_O_3_, with values of ∼143 and ∼119 kPa and fracture strains of 6.4% and 3.1%, respectively. The compression moduli were also slightly lower than for 20% Fe_2_O_3_ G_62_–H_170_–4% GO (Figure S11b). In tensile testing experiments (Figures 3c and S11), the maximum Young’s modulus was 24.2 kPa for a Fe_2_O_3_ concentration of 20% w/w and ∼12 kPa for either 6% or 40% Fe_2_O_3_ G_62_–H_170_–4% GO gels. All samples underwent hardening until fracture, and the fracture strain for 20% Fe_2_O_3_ G_62_–H_170_–4% GO was 13.3%, which was nearly twice that of 6% and 40% Fe_2_O_3_ G_62_–H_170_–4% GO. The fractured samples were allowed to self-heal for 4 h, and the data obtained after healing (dotted lines) were almost identical to the initial curves suggesting good self-healing occurred in all cases. Additionally, while the self-healing studies reported thus far allowed the Fe_2_O_3_-containing gels to recover from breakage for 4 h at room temperature, these nanocomposite worm gels could be reformed (healed) much faster. This was achieved by utilizing the morphological phase transition (worms-to-spheres-to-worms), which occurs for these copolymer gels on cooling to a low temperature in a mold for ∼15 min and returning to room temperature (Figure S12).

### Multifunctional Properties of *x*% Fe_2_O_3_ Nanocomposite Worm Gels

**Figure 4:**
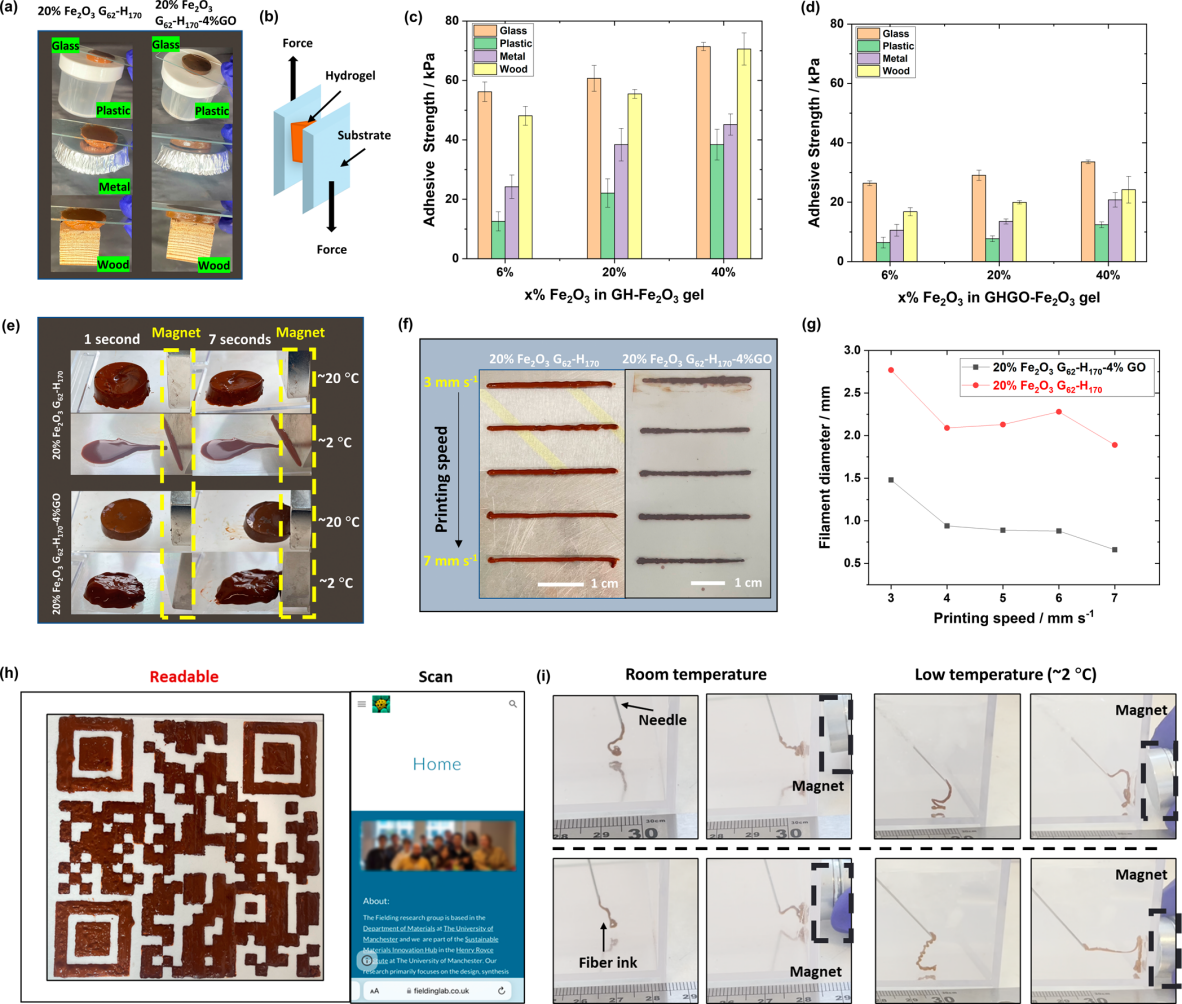
(a) Digital photographs of 20% Fe_2_O_3_ G_62_–H_170_ and 20% Fe_2_O_3_ G_62_–H_170_–4% GO nanocomposite worm gels (brown colored disks) adhered to different substrates (glass, plastic, aluminum, and wood). (b) Schematic diagram of lap-shear test. (c, d) Adhesion strengths measured at room temperature for 20% Fe_2_O_3_ G_62_–H_170_ and 20% Fe_2_O_3_ G_62_–H_170_–4%GO nanocomposite worm gels on glass, plastic, metal, and wood. (e) Digital photographs of the magnetic response of 20% Fe_2_O_3_ G_62_–H_170_ and 20% Fe_2_O_3_ G_62_–H_170_–4%GO nanocomposite worm gels at different temperatures. (f) Digital photographs of nanocomposite gel filaments printed at different printing speeds. (g) Filament diameter as a function of printing speed. (h) 3D-printed QR code (12.5 × 12.5 cm) using 20% Fe_2_O_3_ G_62_–H_170_ copolymer nanocomposite worm gel with a printing speed of 6 mm s^–1^ at room temperature. (i) Digital photographs of 20% Fe_2_O_3_ G_62_–H_170_ (top row) and 20% Fe_2_O_3_ G_62_–H_170_–4% GO (bottom row) nanocomposite worm gels injected under water to form a thread (left images) and attracted by a magnet (right images) at different temperatures.

The nanocomposite worm gels were found to adhere relatively strongly to a variety of surfaces. As shown in Figure 4a, the gels adhered firmly to one side of a glass substrate while maintaining stable adhesion to other substrates, such as plastic, metal, and wood, without any noticeable deformation. Adhesion behavior was further evaluated by lap-shear testing (Figure 4b–d). The gels demonstrated the highest adhesive strengths on glass and wood substrates, with weaker adhesion observed for plastic and metal. For instance, the adhesive strength for 20% Fe_2_O_3_ G_62_–H_170_ to plastic (∼22 kPa) was 3 times smaller than for glass. In addition, with increasing Fe_2_O_3_ concentration, the adhesive strength also increased. However, in contrast to the rheological and mechanical properties described above, the adhesive strength of *x*% Fe_2_O_3_ G_62_–H_170_–4% GO gels was significantly less than that for *x*% Fe_2_O_3_ G_62_–H_170_ samples, implying a decrease in adhesive strength with increasing gel strength.

The presence of Fe_2_O_3_ nanoparticles in these nanocomposite gels should confer magnetic responsive properties. Thus, the magnetic response of 20% Fe_2_O_3_ G_62_–H_170_ and 20% Fe_2_O_3_ G_62_–H_170_–4% GO on a plastic surface at different temperatures was investigated (Figure 4e). At room temperature, the 20% Fe_2_O_3_ G_62_–H_170_–4% GO gel moves directly towards the magnet. However, the 20% Fe_2_O_3_ G_62_–H_170_ gel did not move to the magnet but significantly deformed. This can be attributed to the higher adhesive strength to the plastic for this sample (Figure 4c,d). Interestingly, at low temperatures, the liquid-like 20% Fe_2_O_3_ G_62_–H_170_ sample began flowing to the magnet immediately when applied, whereas the 20% Fe_2_O_3_ G_62_–H_170_–4% GO formulation lost its original shape when cooled but remained in a gel-like state and gradually moved toward to the magnet. These observations are consistent with rheological viscosity measurements (Figure S13) and temperature-dependent oscillatory rheology studies (Figure S9). For example, the viscosity of *x*% Fe_2_O_3_ G_62_–H_170_ samples increased with increasing Fe_2_O_3_ concentrations at room temperature but decreased with increasing Fe_2_O_3_ concentration at ∼2 °C due to the copolymer worm-to-sphere morphological transition which occurs. For the *x*% Fe_2_O_3_ G_62_–H_170_–4% GO formulations, the viscosity at room temperature was much lower than for samples without GO. However, when the temperature was reduced to ∼2 °C, only a partial phase change occurred and nanocomposites transferred from being high-strength gels to soft high-viscosity gels. It is worth noting that for formulations prepared by low-shear mixing, after applying a strong magnet to these samples at low temperatures, the Fe_2_O_3_ particles became separated from the polymer dispersion and moved toward the magnet (Figure S5b). However, for the nanocomposites prepared by high-speed mixing, this did not occur, regardless of temperature (Figure 4e).

Due to their excellent mechanical and shear-recovery properties, these Fe_2_O_3_ nanocomposite worm gels are ideal for 3D gel printing. The diameter of printed filaments based on the printing speed with an 840 μm diameter nozzle at room temperature is shown in Figure 4f–g. The printing speed was varied from 3 to 7 mm s^–1^, and the diameter of both 20% Fe_2_O_3_ G_62_–H_170_ and G_62_–H_170_–4% GO filaments decreased as the printing speed increased, providing more rapid printing times and higher resolutions. However, when the speed was 7 mm s^–1^ for 20% Fe_2_O_3_ G_62_–H_170_, at the end of the filament, there is a noticeable trail left behind, indicating that filament dragging has occurred. For 20% Fe_2_O_3_ G_62_–H_170_–4% GO, when the speed is >6 mm s^–1^, the diameter of the printed filaments can be observed to vary significantly between the front and back portions, whereas the filaments printed at ≤6 mm s^–1^ exhibited greater stability and uniformity. Thus, the optimum printing speed was selected to be 6 mm s^–1^ for both the 20% Fe_2_O_3_ G_62_–H_170_ and 20% Fe_2_O_3_ G_62_–H_170_–4% GO formulations.

To demonstrate the 3D printability of these nanocomposite gels, complex 3-layer QR codes were printed by using 20% Fe_2_O_3_ G_62_–H_170_ and 20% Fe_2_O_3_ G_62_–H_170_–4% GO (Figures 4h and S14). These patterns were printed with high fidelity and no errors, with the QR codes being easily scannable (Video S1). Moreover, the response of these QR codes to low temperature varied based on the composition of the gel used in their preparation. On cooling to 2 °C for 15 min, the 20% Fe_2_O_3_ G_62_–H_170_–4% GO QR code underwent partial degelation but remained readable (Figure S14). However, the originally discrete regions of the QR codes printed using 20% Fe_2_O_3_ G_62_–H_170_ began to lose their shape and merge, rendering them unscannable. In addition, Figure S15 shows thin, 3-layered “UOM” letters which retain their shape and height after printing at room temperature. As expected, cooling the letters printed using 20% Fe_2_O_3_ G_62_–H_170_ to 2 °C for 15 min results in them losing their form as the gels transition to a liquid state, whereas the letters formed from 20% Fe_2_O_3_ G_62_–H_170_–4% GO remain stable.

The injectability of 20% Fe_2_O_3_ G_62_–H_170_ and G_62_–H_170_–4% GO gels underwater was evaluated by using a needle and syringe at various temperatures (Figure 4i). The nanocomposite gels could readily be injected into water without any breakage, even at low temperatures (where the gel strength decreases). Furthermore, upon the introduction of an external magnetic force, the extruded filaments promptly aligned with the direction of the magnet within the water, demonstrating their seamless response to a magnetic field. Remarkably, the filaments remained unbroken even when subjected to magnetic force, allowing them to swiftly move without interruption at both room and low temperatures. Videos S2 and S3 show the injectability of 20% Fe_2_O_3_ G_62_–H_170_ and 20% Fe_2_O_3_ G_62_–H_170_–4% GO underwater at room temperature, respectively and Videos S4 and S5 show the injectability of 20% Fe_2_O_3_ G_62_–H_170_ and 20% Fe_2_O_3_ G_62_–H_170_–4% GO underwater at low temperatures (∼2 °C), respectively. This behavior is analogous to the work of Zhao et al.,^50^ where a water-immiscible coacervate liquid magnetic robot was prepared based on assembled magnetic core–shell nanoparticles. In their work, it was claimed that the reported strategy resolved issues with conventional hydrogels being difficult to apply in biomedicine due to limitations in terms of deformation, e.g., squeezing through capillaries. Compared with these “coacervate-based liquid robots”, the Fe_2_O_3_-containing nanocomposite worm gels reported herein have similar benefits in terms of their stability in both air and water environments, as well as their magnetic response. Additionally, PGMA-*b*-PHPMA has found applications in the biomedical field as an injectable carrier and encapsulation agent.^51^ Consequently, these multifunctional, magnetic nanocomposite gels which can be produced through environmentally friendly, easy-to-operate, and scalable methods clearly have the potential to be used as injectable responsive biomaterials.

### *In Vitro* Biocompatibility Studies

To be considered for use in potential bioapplications, it is important for such materials to also be biocompatible. Indeed, it has previously been shown that PGMA-*b*-PHPMA gels are biocompatible, can be sterilized via filtration at low temperature, and can potentially be used for bioapplications.^27,44,52,53^ Furthermore, GO and Fe_2_O_3_ nanoparticles are also widely studied for use in biomedical applications.^54−57^ However, the combination of these three materials in a single nanocomposite gel may lead to unforeseen biocompatibility issues. Thus, to demonstrate the potential of these nanocomposite gels for bioapplications, initial biocompatibility studies were conducted.

**Figure 5:**
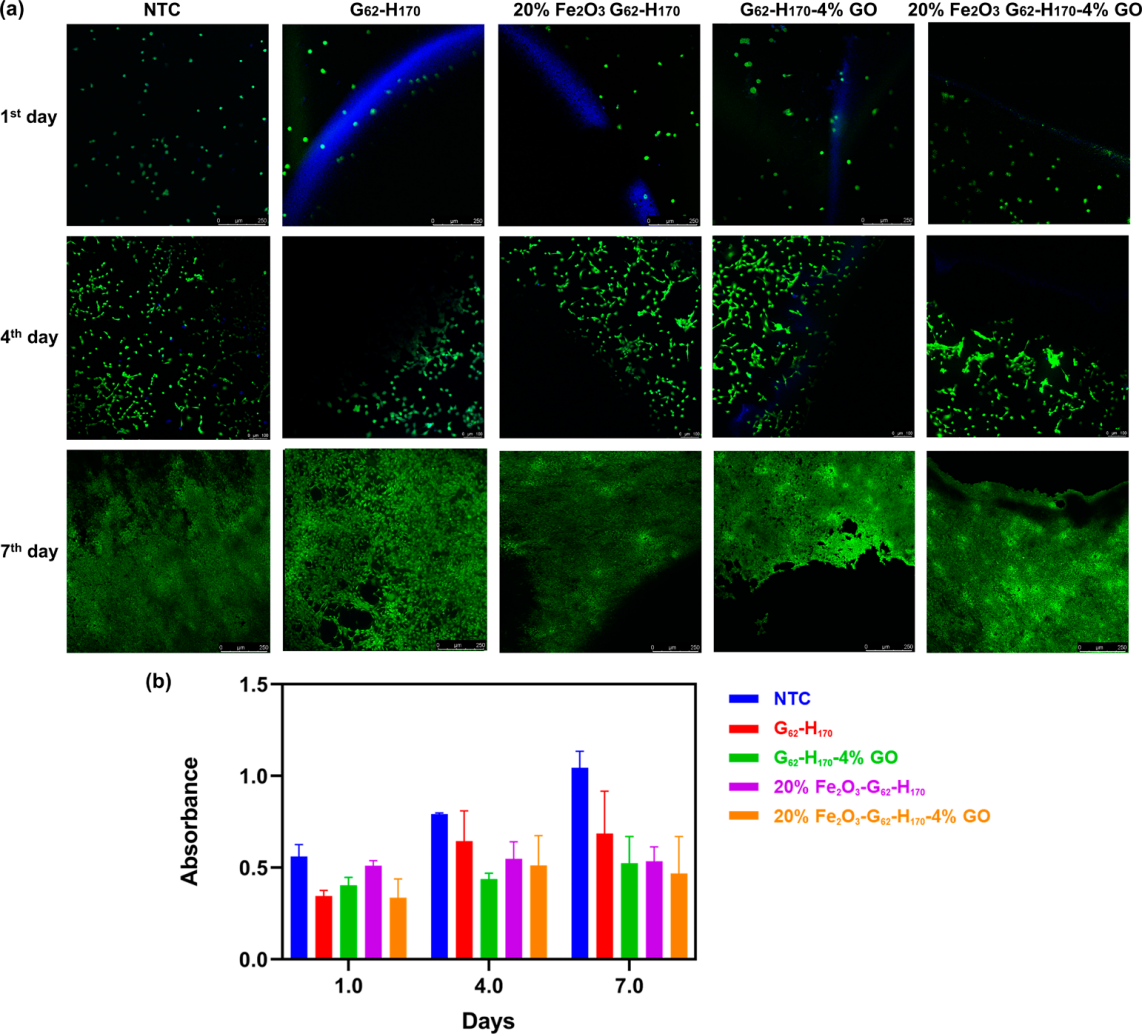
(a) Growth patterns of 3T3 cells at the gel–cell interface for various nanocomposite worm gels and a gel-free control (NTC) at 1, 4, and 7 days since cell seeding. Live cells are highlighted in green, and dark regions are areas where the gel layer prevented laser penetration. The blue coloration apparent in some of the images is due to the edges of the hydrogel. Gel positions were confirmed visually and using the optical mode of the microscope. (b) Metabolic performance (MTS assay) of 3T3 cells, where higher absorbance represents more healthy cells.

The viability of 3T3 cells was investigated in the presence of a gel-free control (NTC) and G_62_–H_170_, G_62_–H_170_–4% GO, 20% Fe_2_O_3_ G_62_–H_170_, and 20% Fe_2_O_3_ G_62_–H_170_–4% GO gels (Figure 5a). In all cases, Live/Dead assays showed that all cells present were stained green (live) over the course of 7 days. In addition, the live cell density still increased strongly after 7 days for all samples studied. However, it is worth noting that neither live nor dead cells were observed on the gels themselves (dark regions of the images). This is due to the cells not adhering strongly to the gels, which subsequently led to their displacement during washing as part of the assay. Nevertheless, cell growth was apparent in all cases, and therefore, it can be concluded that the nanocomposite gel composition did not appear to significantly influence cell viability.

In the MTS assay (Figure 5b), all samples studied exhibited increased cell metabolic activities on the fourth day. However, the rate of increase in metabolic activity by the seventh day for cells incubated with the copolymer and nanocomposite gels was lower than for the gel-free control. This can potentially be attributed to the fact that the presence of gel in the sample imposes spatial restraints on cell growth, and lower activities are recorded. On inspecting the differences in activity between each gel composition, there are minimal differences between gels formulated with and without GO and Fe_2_O_3_ and therefore the addition of these nanomaterials seems to have no significant adverse impact on the biocompatibility of the copolymer gels. Thus, they have the potential to be used in medical applications or as bio/wearable electronics.

### Strain and Temperature Sensing

Given that these nanocomposite worm gels contain NaCl, they have a degree of electrical conductivity due to this electrolyte. While GO, Fe_2_O_3_, and PGMA-*b*-PHPMA are not electrically conductive, the above studies demonstrate that these nanocomposite worm gels physically respond to temperature and stress variations. Notably, the conductivity of the gels also changes in response to these variations, making them potentially useful as sensors. Furthermore, since they can be actuated under the effect of a magnetic force, they can potentially act as soft robots and function as an on/off switch for controlling circuitry.

A relatively simple example of this is provided in Figure S16 where an unconnected electrical circuit is positioned on a plastic plate and nanocomposite gels are placed on its surface. The movement of the gels is controlled without contact using a magnet, initially toward the right and then forward to reach the designated position to establish a connection with the circuit, resulting in the illumination of an LED. Videos S6 and S7 show this magnetically driven motion for 20% Fe_2_O_3_ G_62_–H_170_ and 20% Fe_2_O_3_ G_62_–H_170_–4%GO nanocomposite worm gels. To further understand the electrical behavior of these nanocomposite gels, the performance of the 20% Fe_2_O_3_ G_62_–H_170_ and G_62_–H_170_–4% GO samples as strain and temperature sensors was investigated.

**Figure 6:**
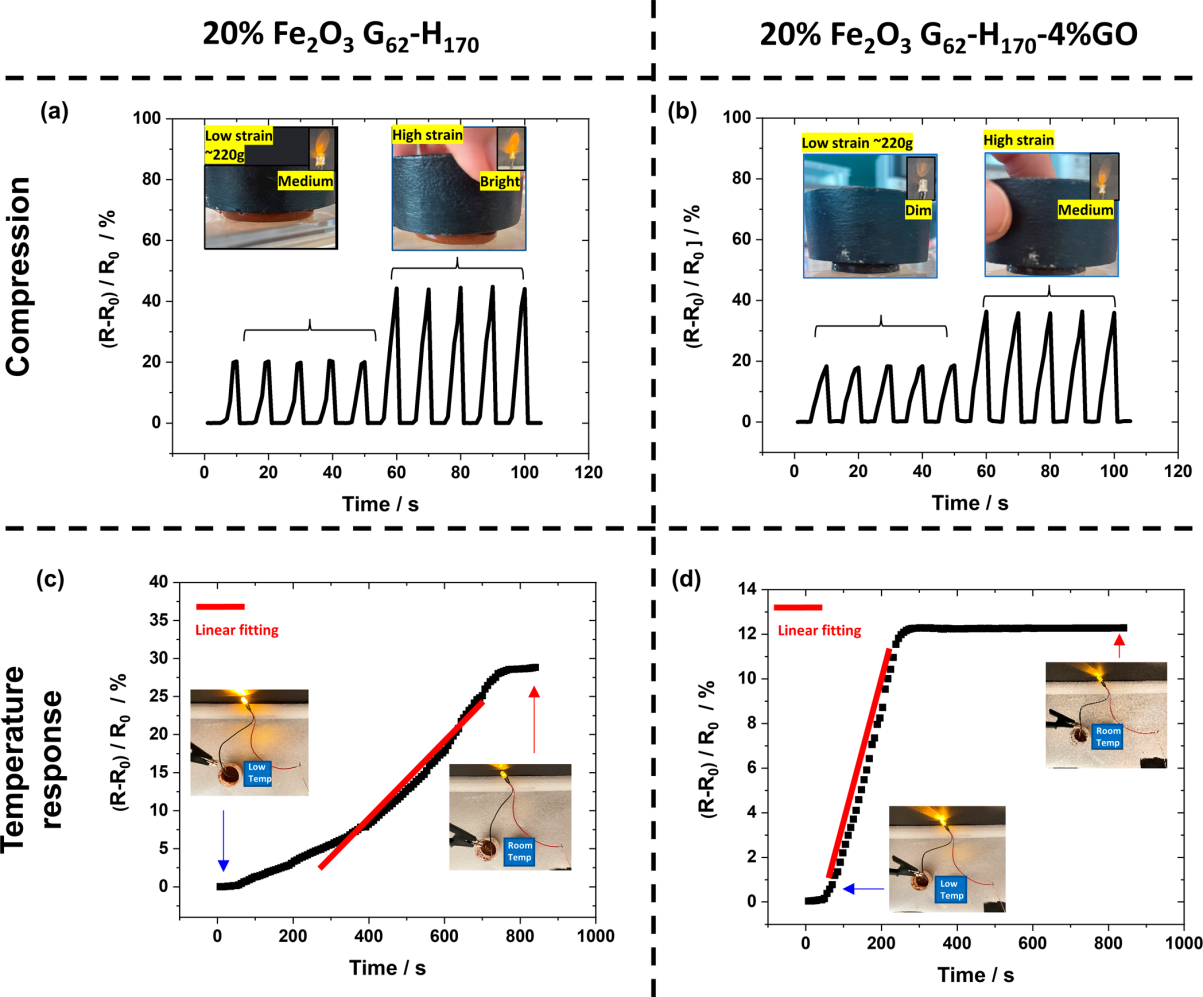
Variation in relative resistance for (a) 20% Fe_2_O_3_ G_62_–H_170_ and (b) 20% Fe_2_O_3_ G_62_–H_170_–4% GO nanocomposite worm gels over 5 low compression stress cycles and 5 high compression stress cycles. Digital photograph insets show the gels being compressed and LED brightness during compression. (c, d) Relative resistance for 20% Fe_2_O_3_ G_62_–H_170_ and 20% Fe_2_O_3_ G_62_–H_170_–4% GO nanocomposite gels, respectively, as the gels warmed from 2 °C to room temperature. The insets show digital photographs of LED brightness change during these experiments.

Figure 6a,b shows the variation in relative electrical resistance and LED light intensity of these gels through numerous compression-relaxation cycles. During this test, cylindrical gels were placed between two copper wires adhered to carbon tabs and connected an electrical circuit containing an LED. A constant voltage was applied to the system, and the change in relative resistance [(*R* – *R*_0_)/*R*_0_, where *R*_0_ is the initial resistance before compression and *R* is the measured resistance] was recorded during manual compression/relaxation cycles. After several low compression cycles (10 s each cycle), the applied compression force was increased for further cycles (10 s each cycle). On application of a compression force to the composite gel, the value of resistance (*R*_0_) is relatively low, and the LED brightened. When the pressure was released, the composite gel returned to its original state, the resistance significantly increased and the LED dimmed. The relative resistance change upon compression was rapid and was consistent with the amount of compression applied, indicating the potential for good device stability. When the two gels studied were compared, the relative resistance changes for the 20% Fe_2_O_3_ G_62_–H_170_ gel were slightly larger than for the 20% Fe_2_O_3_ G_62_–H_170_–4% GO gel, particularly when a higher compressive force was applied. On removal of the compressive force, both nanocomposite worm gels relaxed almost immediately, and the resistance repeatedly recovered back to *R*_0_ after each cycle.

The conductivity of Fe_2_O_3_-containing nanocomposite gels was also found to be temperature-dependent. Figure 6c,d shows the relative resistance change as a function of temperature for 20% Fe_2_O_3_ G_62_–H_170_ and G_62_–H_170_–4%GO nanocomposite worm gels as they were allowed to warm from ∼2 °C to room temperature (with *R*_0_ defined as the resistance at low temperature in this study). In these cases, the mobility of free ions (from NaCl) decreases with increasing temperature due to the increase in gel strength (Figures 2g and 3g), which subsequently leads to a decrease in conductivity (an increase in resistance). Thus, on warming these gels, the brightness of the connected LED diminishes. For 20% Fe_2_O_3_ G_62_–H_170_, the sample was in a liquid state at 2 °C and thus the change in resistance on returning to room temperature was larger (27%) than for the GO-containing gel (12%), which did not fully undergo gelation on cooling (leading to a larger value of R_0_). 20% Fe_2_O_3_ G_62_–H_170_–4% GO gel had a faster “recovery time” than the 20% Fe_2_O_3_ G_62_–H_170_ sample, with the former taking ∼4.5 min for the resistance to equilibrate and the latter taking ∼12 min. This is likely due to the non-GO-containing sample requiring more sphere-to-sphere fusion to occur for this gel to reform compared to the GO-containing sample which did not fully degel.

In summary, these Fe_2_O_3_-containing nanocomposite worm gels exhibit tunable rheological and mechanical properties (based on formulation and temperature), are magnetic, can be 3D-printed, and have electrical properties which vary as a function of compression and temperature. The straightforward demonstrations of 3D printability, magnetic-, electrical- and thermo-response reported herein show that these gels have the capacity to move, deform, or transition into a liquid-like state and it is notable that the range of Young’s moduli demonstrated is similar to many biological tissues including muscle.^58^ This distinctive amalgamation of smart attributes opens up numerous possibilities for their utilization in a diverse range of applications, such as soft robotics, responsive information protection, as sensors, in medical applications, or as bio/wearable electronics.^6,59,60^

## Conclusions

The incorporation of magnetic nanoparticles into block copolymer worm gels facilitates the formation of magnetic hydrogels with a wide range of functional properties. Self-healing and temperature-responsive behavior are imparted by the worm gel matrix, which is synthesized via RAFT aqueous dispersion polymerization, and enhanced mechanical strength is imparted by the inclusion of GO. The addition of Fe_2_O_3_ nanoparticles is achieved through a new mixing method for these types of materials using a high-speed mixer at low temperature, resulting in further improvements in mechanical properties and the addition of magnetic functionality. Thus, optimized 20% Fe_2_O_3_ G_62_–H_170_ and 20% Fe_2_O_3_ G_62_–H_170_–4% GO gel formulations are injectable, making them suitable for 3D printing in nanocomposite engineering^4,8,61−63^ and as injectable magnetic-responsive biomaterials.^50,64−68^ They also retain their self-healing and temperature-responsive properties, show promising biocompatibility, and have been demonstrated as potential strain and temperature sensors. This unique combination of multifunctional properties means that this class of materials has the potential for future applications in areas including biomedical, electronics, and other related fields.^69,70^

## Supporting Information Available

Additional experimental materials and methods; supporting data tables; reaction schemes; ^1^H NMR spectra and GPC chromatograms; additional digital photographs of samples; tensile, compression, and rheological testing data; and TEM images (PDF)
Easily scannable QR codes (Video S1) (MP4)
Injectability of 20% Fe_2_O_3_ G_62_–H_170_ underwater at room temperature (Video S2) (MP4)
Injectability of 20% Fe_2_O_3_ G_62_–H_170_–4% GO underwater at room temperature (Video S3) (MP4)
Injectability of 20% Fe_2_O_3_ G_62_–H_170_ underwater at low temperature (Video S4) (MP4)
Injectability of 20% Fe_2_O_3_ G_62_–H_170_–4% GO underwater at low temperature (Video S5) (MP4)
Magnetically driven motion for 20% Fe_2_O_3_ G_62_–H_170_ nanocomposite worm gels (Video S6) (MP4)
Magnetically driven motion for 20% Fe_2_O_3_ G_62_–H_170_–4%GO nanocomposite worm gels (Video S7) (MP4)
