# Supplementary material for: Multifunctional Self-Assembled Block Copolymer/Iron Oxide Nanocomposite Hydrogels Formed from Wormlike Micelles
Source: ACS Appl Mater Interfaces. 2024 Apr 9;16(16):21197–209. doi: 10.1021/acsami.4c03007 (PMC11056933; doi:10.1021/acsami.4c03007)
Supplement: Supplementary file 1 — am4c03007_si_001.pdf [file am4c03007_si_001.pdf]

Supporting Information:

Multifunctional self-assembled block copolymer/iron oxide nanocomposite hydrogels formed from wormlike micelles.

Qi Yue,[a,b] Shiyu Wang,[a,b] Samuel Jones,[a,b,c] and Lee A. Fielding[a,b]*

a. *Department of Materials, School of Natural Sciences, University of Manchester, Oxford Road, Manchester, M13 9PL, U.K.*

b. *Henry Royce Institute, The University of Manchester, Oxford Road, Manchester, M13 9PL, U.K.*

c. *School of Chemistry, University of Birmingham, Edgbaston, Birmingham, B15 2TT, U.K.*

** Corresponding author: lee.fielding@manchester.ac.uk*

Additional experimental materials and methods

Materials. Glycerol monomethacrylate (GMA) was kindly donated from GEO Specialty Chemicals (U.K.), 4-Cyano-4-(2-phenylethane sulfanylthiocarbonyl) sulfanylpentanoic acid (PETTC) was prepared in-house using previously published methods.[1, 2] 2-Hydroxypropyl methacrylate (HPMA, 97%) was purchased from Alfa Aesar (U.K.). Ethanol (95%) was purchased from Fisher Scientific (UK) and used as received. Graphene oxide (GO) dispersion was purchased form Graphena (Spain) and purified before use. Deionized water was used in all experiments.

Synthesis of poly(glycerol monomethacrylate) (PGMA) *via* RAFT solution polymerization. PGMA was synthesized by RAFT polymerization in ethanol at 70 °C (Figure S1) using a procedure which has been widely reported in the literature.[3-6] For PGMA with a degree of polymerization of 62 (G_{62}), GMA monomer (20 g, 124 mmol) and PETTC RAFT agent (0.68 g, 2.0 mmol) were weighed into a 250 mL round bottomed flask and purged with N_2 for 30 min. 4,4-azobis(4-cyanovaleric acid) (ACVA) initiator (0.11 g, 0.4 mmol, PETTC/ACVA molar ratio = 5:0) and anhydrous ethanol (20.8 g, previously purged with N_2 for 20 min) were then added, and the resulting yellow solution was degassed for a further 15 min while stirring to form a homogeneous solution. The flask was subsequently sealed and immersed in an oil bath set at 70 °C. After 120 min, the polymerization was quenched by immersion in an ice bath and opening to air. The final degree of polymerization (DP) was 62, as determined by ^{1}H-NMR analysis (Figure S2a) using D_2O. The polymer was purified by dialysis (MWCO = 3500 g mol^{-1}) against deionized water and freeze-dried to form a yellow powder. DMF GPC analysis indicated an M_n of 7300 g mol^{-1} and an M_w/M_n of 1.13 (Figure S3).

Preparation of PGMA-b-PHPMA worm gels *via* RAFT aqueous dispersion polymerization of 2-hydroxypropyl methacrylate (HPMA). The preparation of PGMA-b-PHPMA worm-gels by RAFT dispersion polymerization (Figure S1a) has been reported extensively in the literature.[3, 7-9] A typical protocol for the synthesis of a $PGMA_{62}$-b-$PHPMA_{170}$ (G_{62}-H_{170}) worm gel is as follows. $PGMA_{62}$ macro-CTA (6.0 g, 0.58 mmol) and HPMA monomer (14.3 g, 99.3 mmol; target DP = 170) were weighed into a 100 mL round bottomed flask and purged with N_2 for 20 min. ACVA was added (54.57 mg, 0.195 mmol, CTA/ACVA molar ratio = 3.0) and purged with N_2 for a further 5 min. Deionized water (81.48 mL, producing a 20.0 % w/w aqueous solution), which had been previously purged with N_2 for 30 min, was then added and the solution was purged for a further 5 min prior to immersion in an oil bath set at 70 °C. The reaction was stirred for 3 h before the HPMA polymerization was quenched by exposure to air. The product was a soft free-standing gel (Figure 2b, inset). The absence of signals owing to the vinyl protons of the HPMA monomer in the ^{1}H NMR spectrum indicated that the polymerization had attained more than 99% conversion (Figure S2b). DMF GPC analysis indicated an M_n of 19 000 g mol^{-1} and an M_w/M_n of 1.10 (Figure S3).

Preparation of GO-containing PGMA-b-PHPMA nanocomposite worm gels by *in situ* RAFT polymerization. The preparation of GO-containing PGMA-b-PHPMA worm gels has been reported previously.[10] Herein, $PGMA_{62}$-b-$PHPMA_{170}$-x% GO nanocomposite worm gels were prepared by *in situ* RAFT aqueous dispersion polymerization of HPMA in the presence of a $PGMA_{62}$ macromolecular chain-transfer agent and GO (Figure S1b). Specifically, the preparation of a 20 % w/w $PGMA_{62}$-b-$PHPMA_{170}$ worm gel containing 4 % w/w GO, based on copolymer, is as follows. $PGMA_{62}$ macro-CTA (5.0 g, 0.48 mmol) and HPMA monomer (11.9 g, 82.8 mmol; target DP = 170) were weighed into a 250 mL round bottomed flask and purged with N_2 for 20 min. 4,4-Azobis(4-cyanovaleric acid) (ACVA) was added (45.5 mg, 0.16 mmol, CTA/ACVA molar ratio = 3.0) and purged with N_2 for a further 5 min. GO dispersion 45.1 mL (15 mg mL^{-1}) and deionized water (25 mL, producing a 20.0 % w/w dispersion in total), which had been previously purged with N_2 for 30 min, was then added and the dispersion was purged for a further 5 min prior to

immersion in an oil bath set at 70 °C. The reaction was stirred for 3.5 h before the polymerization was quenched by exposure to air. The product was a soft free-standing gel (Figure 3b, inset).

^{1}H NMR spectroscopy. ^{1}H NMR spectra were recorded on a Bruker Avance III 400MHz spectrometer with 128 scans averaged per spectrum at 25 °C. $PGMA_{62}$ was dissolved in D_2O, and $PGMA_{62}$-b-$PHPMA_{170}$ copolymers were freeze-dried and dissolved in DMSO-d6 prior to analysis. $PGMA_{62}$-b-$PHPMA_{170}$-4%GO was dissolved in methanol first, then GO flakes were removed using centrifugation. The resulting copolymer was dissolved in DMSO-d6 after being freeze-dried.

Gel permeation chromatography (GPC). 0.5 % w/w polymer solutions were prepared in DMF containing DMSO (10 µL mL^{-1}) as a flow-rate marker. GPC measurements were conducted using HPLC-grade DMF eluent containing 10 mM LiBr at 60 °C at a flow rate of 1.0 mL min^{-1}. An Agilent Technologies 1260 Infinity GPC / SEC system fitted with two Polymer Laboratories PL gel 5µm Mixed C columns connected in series and a refractive index detector was used to assess molar mass distributions using polystyrene calibration standards.

Transmission electron microscopy (TEM). Dispersions were diluted to 0.1 % w/w at 20 °C prior to staining. 3 µL was then placed onto 400 mesh carbon-coated cooper grids for 90 min and carefully blotted with filter paper to remove excess dispersion. The samples were stained in the vapor space above RuO_4 solution for 7 min at room temperature.[11] Imaging was performed using a FEI Tecnai G2 20 instrument connected to a Gatan 1k CCD camera at an accelerating voltage of 200 kV.

Supporting data tables

Table S1. Summary of oscillatory rheology data for iron oxide containing nanocomposite worm gels.

Entry	Composition	G' in LVR (kPa) [a]	CGT [b]		G' after temperature change cycle (kPa)[c]	Recovery efficiency (%) [d]
			Cooling (°C)	Heating (°C)		
1	6% Fe_2O_3 G_{62}-H_{170}	5.8 ± 0.3	5	4	5.2 ± 0.3	87.2
2	20% Fe_2O_3 G_{62}-H_{170}	7.9 ± 0.5	2	3	7.6 ± 0.9	95.1
3	40% Fe_2O_3 G_{62}-H_{170}	4.8 ± 0.6	9	8	4.4 ± 0.1	81.6
4	6% Fe_2O_3 G_{62}-H_{170}-4%GO	27.8 ± 2.4	N/A	3	25.1 ± 1.5	94.5
5	20% Fe_2O_3 G_{62}-H_{170}-4%GO	31.5 ± 1.7	N/A	N/A	31.3 ± 0.8	99.4
6	40% Fe_2O_3 G_{62}-H_{170}-4%GO	23.0 ± 2.2	6	7	20.2 ± 1.4	90.8

[a] LVR: Linear viscoelastic region.

[b] CGT: Critical gelation temperature: cross-over point of G' and G''.

[c] Determined by measuring G' after the temperature was varied from 20 °C to 2 °C to 20 °C s a 2 h equilibration at each temperature for 6 cycles and a final cycle which was equilibrated at 20 °C for 12 h.

[d] Calculated by dividing the final G' at low strain (0.2%) by the G' of the original sample at low strain during the rheology shear-thinning recovery test.

Table S2. Summary of tensile test data for iron oxide containing nanocomposite worm gels.

Entry	Composition	Young's modulus (kPa)	Fracture strain (%)	Compressive modulus (kPa)
1	6% Fe_2O_3 G_{62}-H_{170}	8.1 ± 3.8	7.7 ± 0.2	3.9 ± 1.3
	After healing [a]	7.8 ± 4.4	7.6 ± 0.4	4.0 ± 0.7
2	20% Fe_2O_3 G_{62}-H_{170}	18.3 ± 9.7	11.9 ± 0.3	4.5 ± 2.3
	After healing [a]	19.5 ± 7.8	11.9 ± 0.2	4.5 ± 2.7
3	40% Fe_2O_3 G_{62}-H_{170}	7.2 ± 1.8	3.4 ± 0.8	1.9 ± 0.8
	After healing [a]	5.5 ± 3.4	3.5 ± 1.2	2.1± 0.5
4	6% Fe_2O_3 G_{62}-H_{170}-2% GO	105.3 ± 7.2	7.6 ± 2.2	9.0 ± 2.2
	After healing [a]	93.7 ± 12.4	7.6 ± 1.7	8.3 ± 3.6
5	20% Fe_2O_3 G_{62}-H_{170}-4% GO	356.4 ± 22.2	13.3 ± 3.5	11.4 ± 1.8
	After healing [a]	345.7 ± 29.6	12.4 ± 2.7	10.9 ± 2.4
6	40% Fe_2O_3 G_{62}-H_{170}-6% GO	54.5 ± 13.1	6.5 ± 1.5	7.6 ± 1.6
	After healing [a]	48.7 ± 15.2	6.4 ± 2.1	7.3 ± 2.0

[a] Samples were re-molded after failure for 4h at room temperature and measured again with same parameters.

Supporting Figures

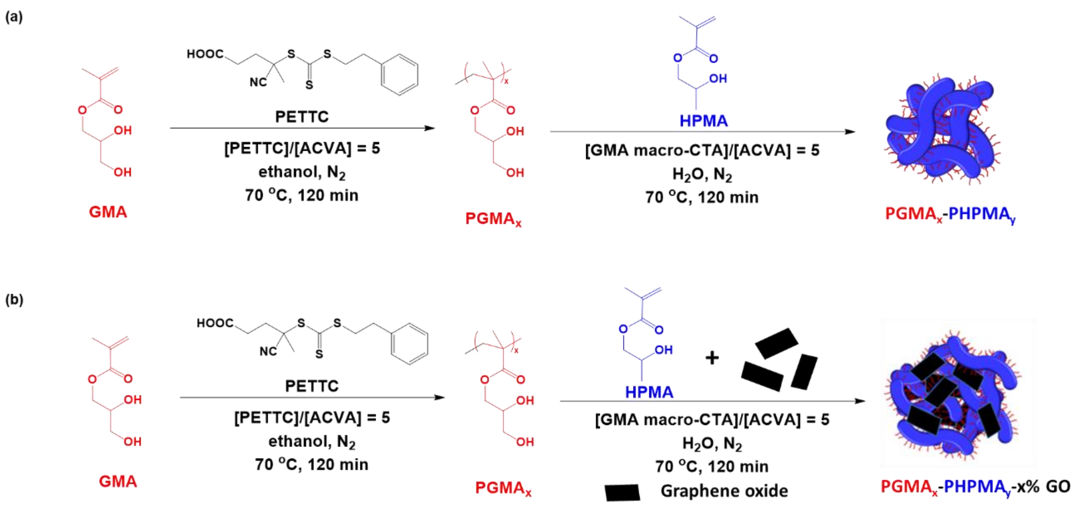

Figure S1. Synthesis of $PGMA_x$ macromolecular chain-transfer agent (macro-CTA) *via* RAFT solution polymerization in ethanol, and (a) $PGMA_x$-b-$PHPMA_y$ and (b) $PGMA_x$-b-$PHPMA_y$-GO *via* RAFT *in-situ* dispersion polymerization in water at 70°C.

(a)

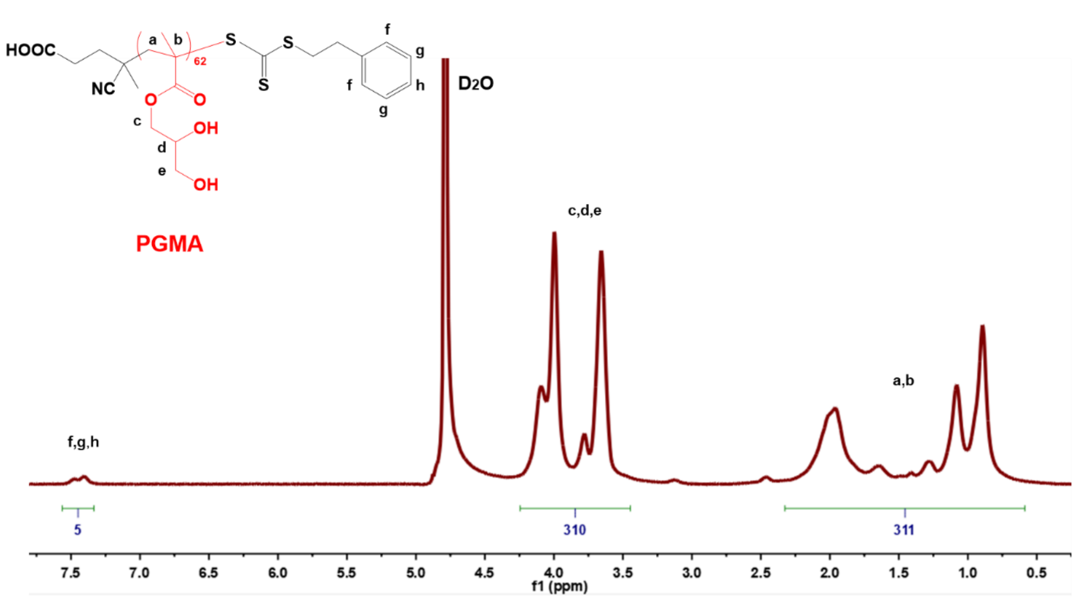

(b)

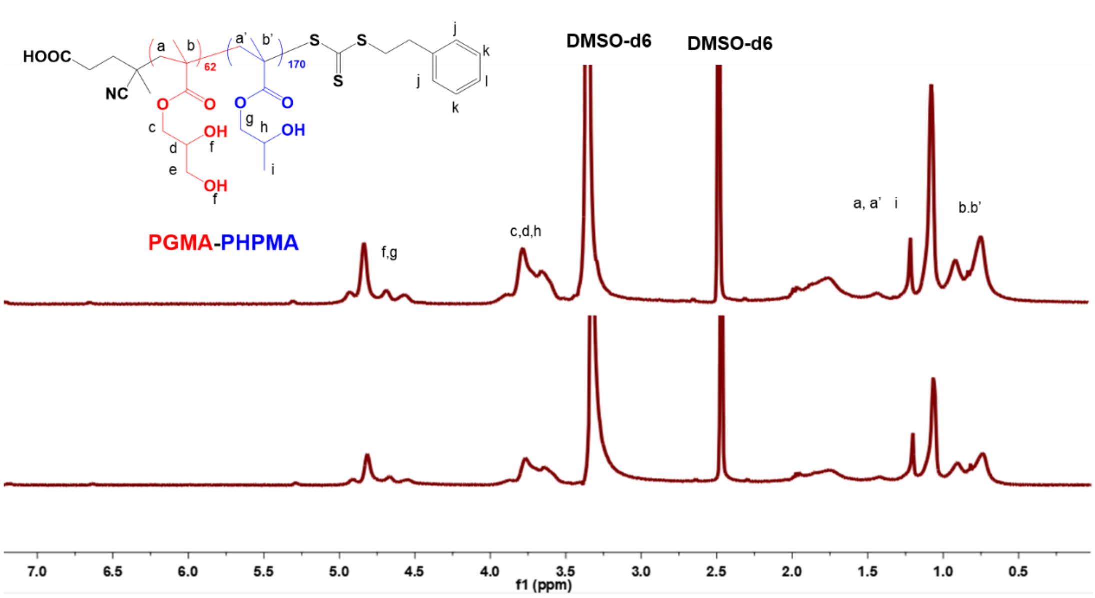

Figure S2. Assigned ^{1}H NMR spectra of (a) GMA_{62} macro-CTA in D_2O and (b) top: $PGMA_{62}$-b-$PHPMA_{170}$ diblock copolymer, bottom: $PGMA_{62}$-b-$PHPMA_{170}$-4% GO in DMSO-d6.

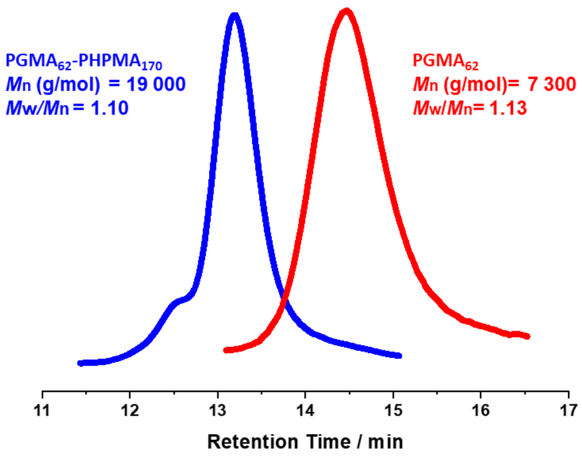

Figure S3. DMF GPC data recorded for $PGMA_{62}$ macro-CTA, and $PGMA_{62}$-b-$PHPMA_{170}$. M_n and M_w/M_n values were determined using polystyrene calibration standards.

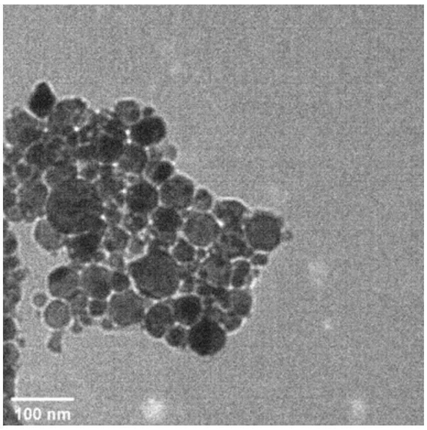

Figure S4. TEM images of iron oxide particles. The sample was diluted to 0.1 % w/w before being deposited on to carbon-coated TEM grids at room temperature.

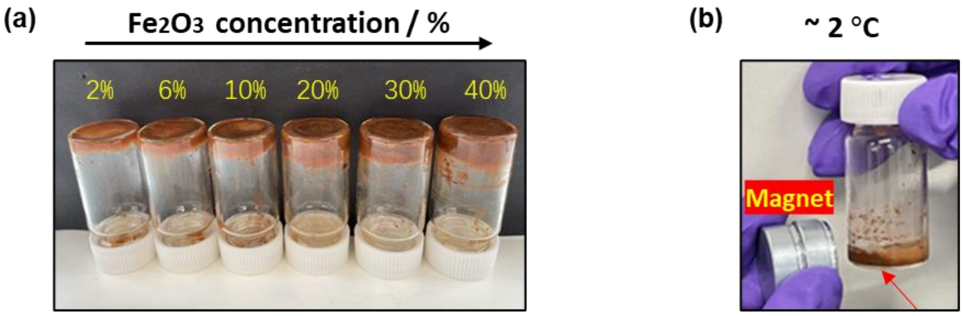

Figure S5. Photographs for (a) various concentrations of Fe_2O_3 containing $PGMA_{62}$-b-$PHPMA_{170}$ samples prepared by low shear mixing by hand. (b) 20% Fe_2O_3 $PGMA_{62}$-b-$PHPMA_{170}$ at ~2 °C held next to a strong magnet. Most of the iron oxide becomes separated from the copolymer under the application of a magnetic force (see red arrow).

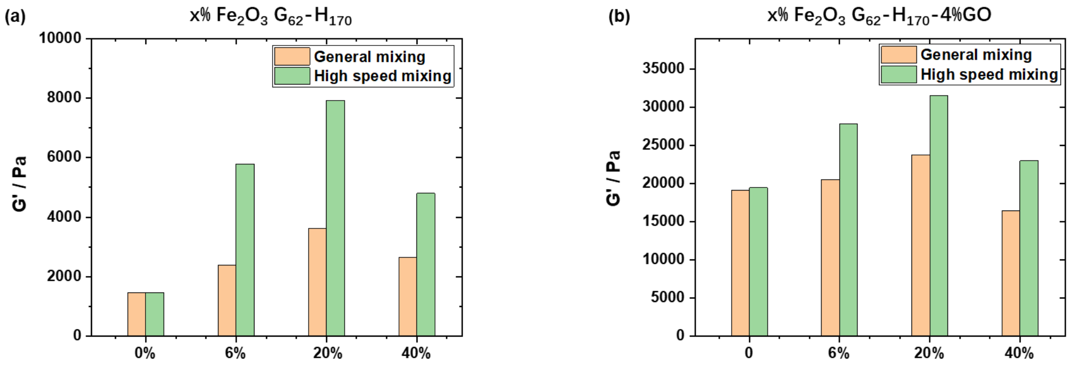

Figure S6. Average storage moduli measured in the linear viscoelastic region for (a) Fe_2O_3 containing G_{62}-H_{170} and (b) Fe_2O_3 containing G_{62}-H_{170}-4% GO dispersions studied using low-shear ('general') and high-speed mixing. All measurements were conducted at 10 rad s^{-1} at a controlled temperature of 25 °C.

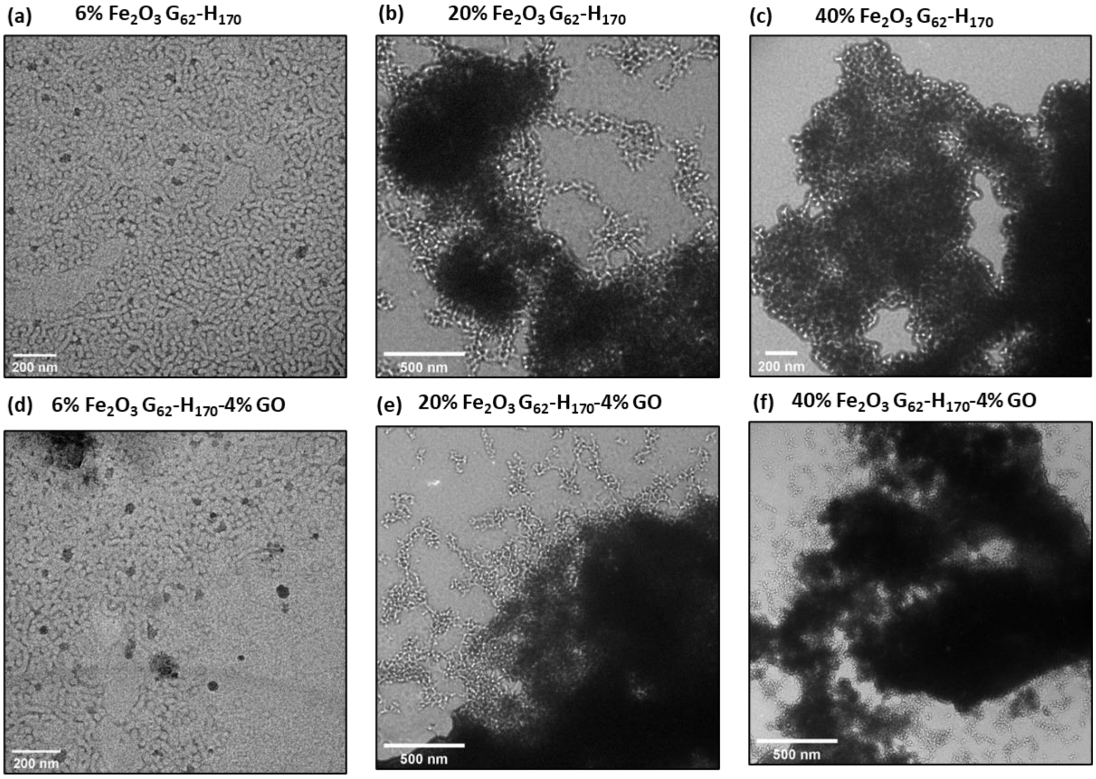

Figure S7. TEM images of: (a-c) x% Fe_2O_3 containing G_{62}-H_{170} nanocomposite worm gels; (d-f) x% Fe_2O_3 containing G_{62}-H_{170}-4% GO nanocomposite worm gels. All TEM samples were diluted to 0.1 % w/w before being deposited on to carbon-coated TEM grids and were stained in the vapor space above RuO_4 solution for 7 min at room temperature.

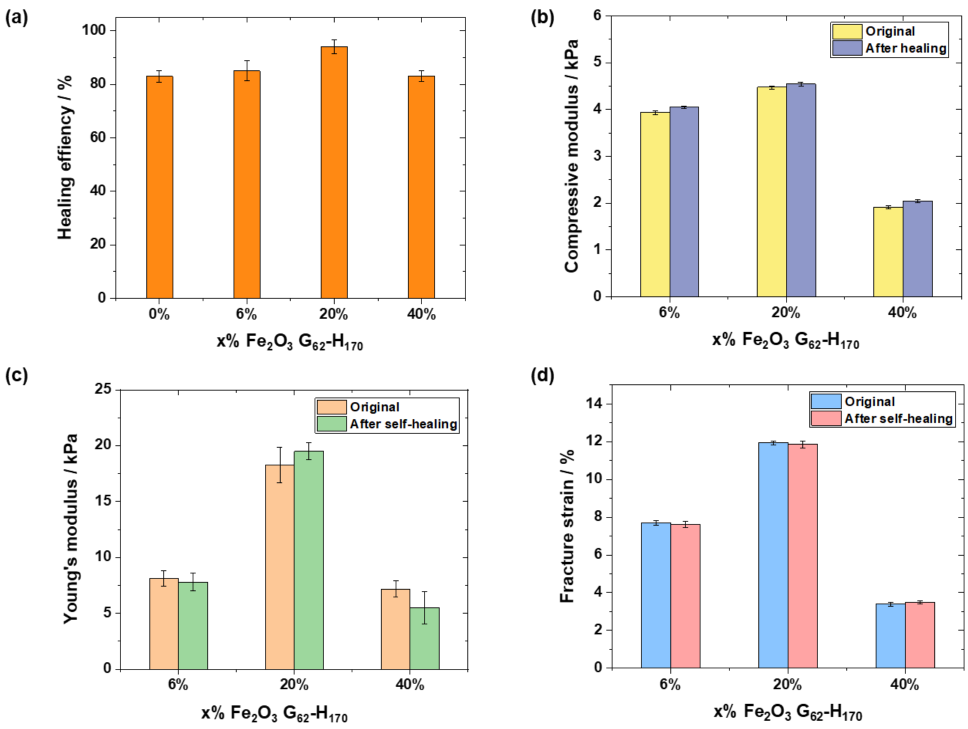

Figure S8. Comparisons of properties for x% Fe_2O_3 G_{62}-H_{170} nanocomposite worm gels (x = 6%, 20% and 40% based on copolymer). (a) Healing efficiency calculated from oscillatory rheology recovery experiments; (b) Compressive moduli; (c) Young's moduli; and (d) fracture strain.

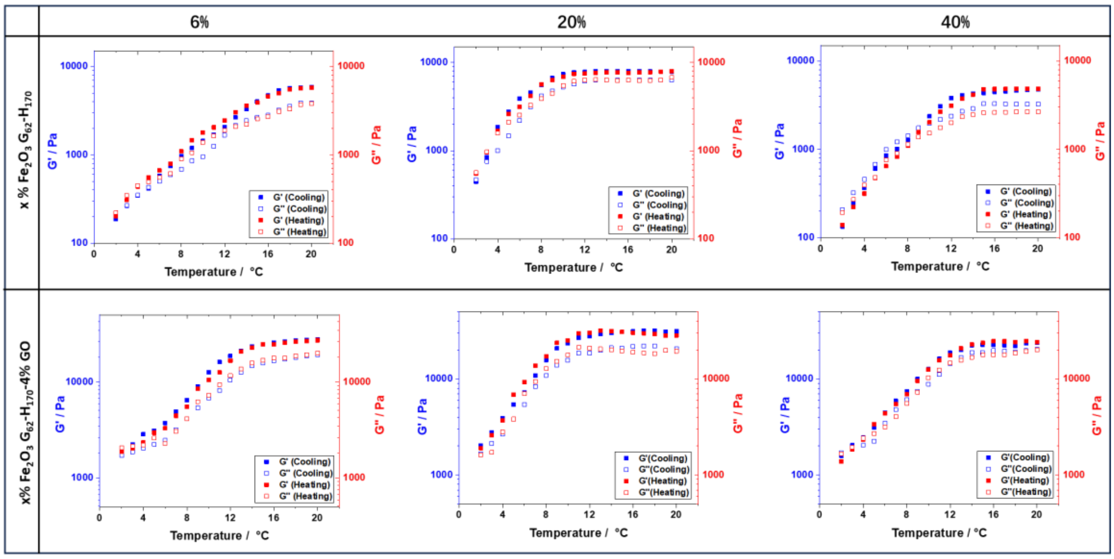

Figure S9. Temperature-dependent oscillatory rheology studies for iron oxide containing nanocomposite worm gels. The temperature was varied from 20 °C to 2 °C to 20 °C in 1 °C steps with 3 minutes equilibration at each step. All measurements were conducted at an angular frequency of 10 rad s^{-1} and applied strain amplitude of 1.0 %.

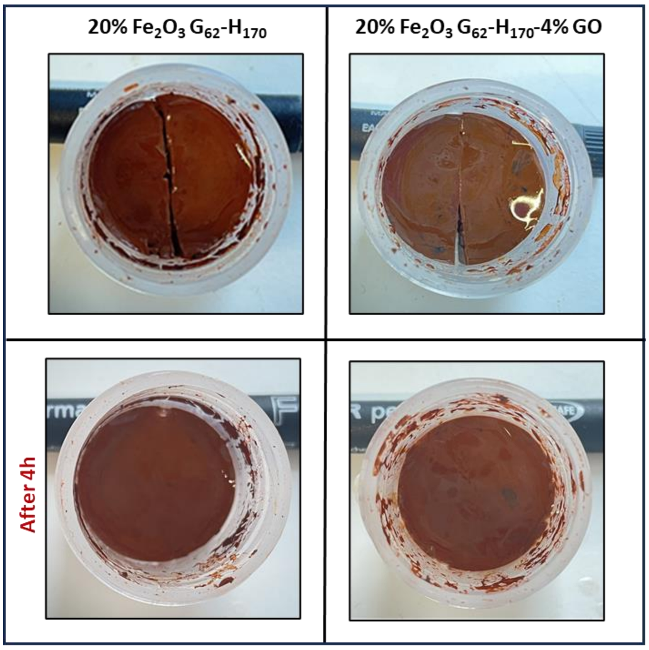

Figure S10. Photographs of 20% Fe_2O_3 containing G_{62}-H_{170} and G_{62}-H_{170}-4% GO nanocomposite worm gels. Upper images show samples being cut and the lower images are after self-healing for 4 h at room temperature.

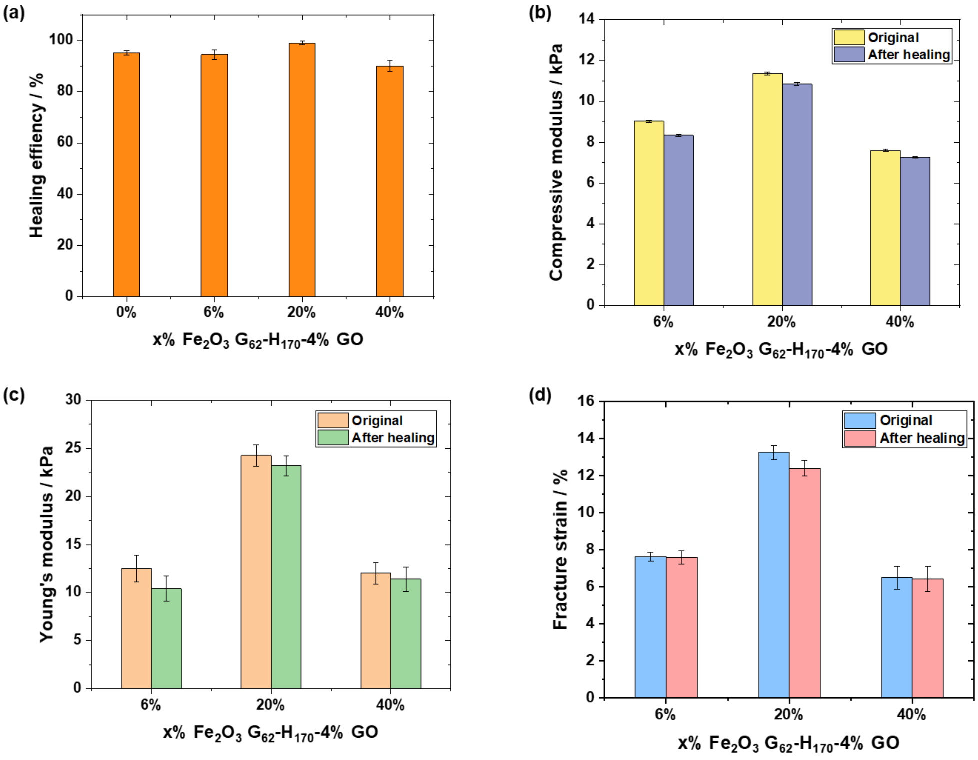

Figure S11. Comparisons of properties for x% Fe_2O_3 G_{62}-H_{170}-4% GO nanocomposite worm gels (x = 6%, 20% and 40% based on copolymer). (a) Healing efficiency calculated from oscillatory rheology recovery experiments; (b) Compressive moduli; (c) Young's moduli; and (d) fracture strain.

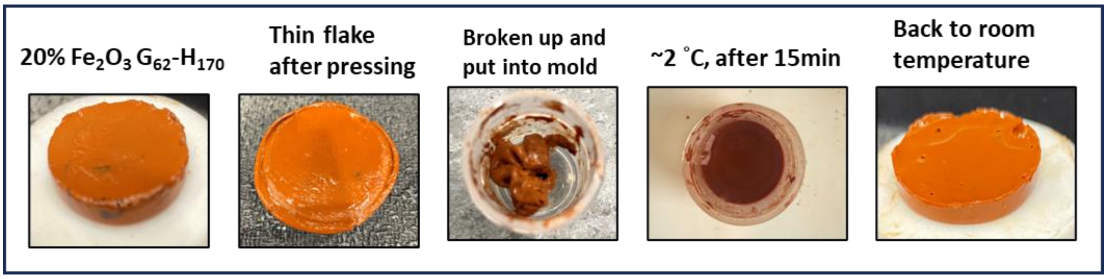

Figure S12. Digital photographs of self-healing process *via* cooling for 20% Fe_2O_3 G_{62}-H_{170} nanocomposite worm gel.

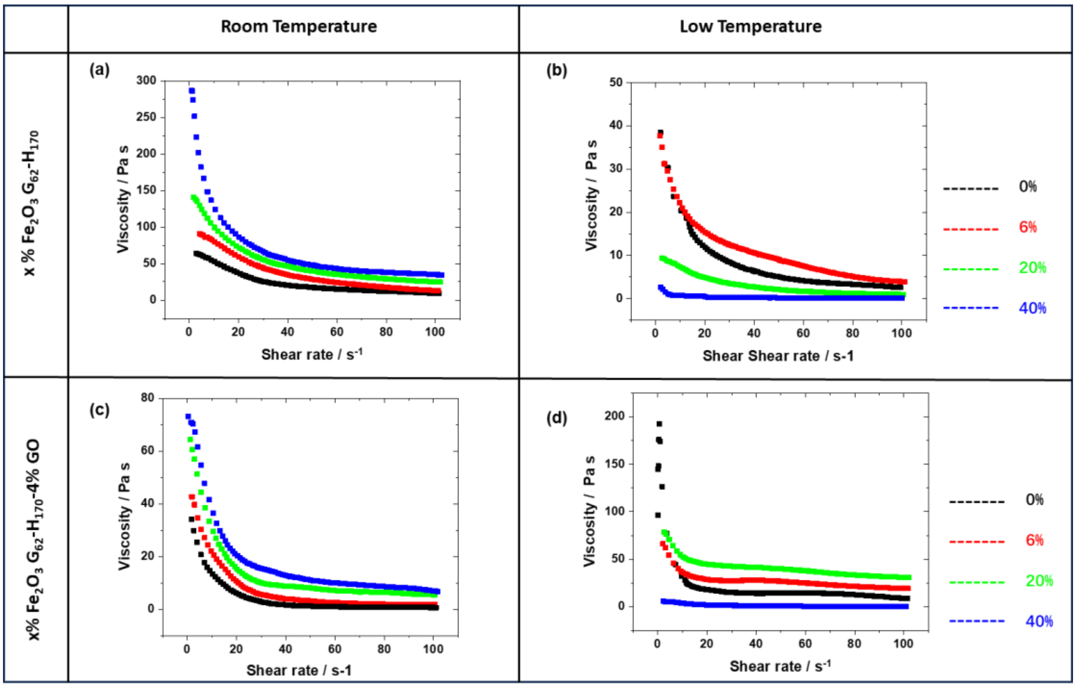

Figure S13. Viscosity of (a, b) 20% Fe_2O_3 G_{62}-H_{170} and (c, d) 20% Fe_2O_3 G_{62}-H_{170}-4% GO nanocomposite gels as a function of shear rate (s^{-1}) at room temperature and low temperature (2°C).

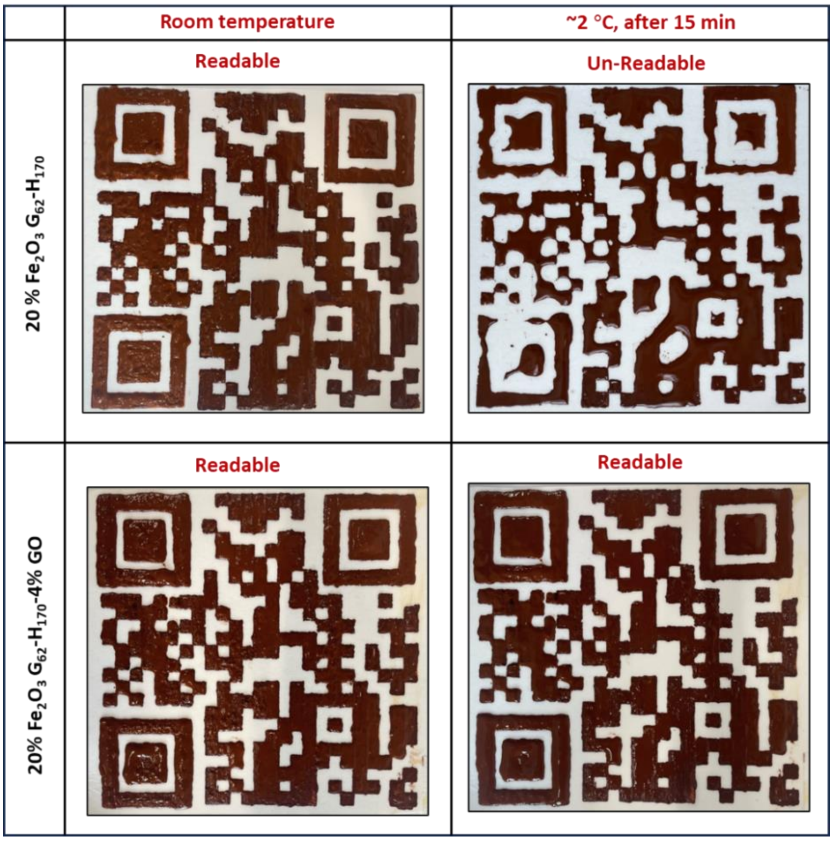

Figure S14. 3D-printed QR codes (each 12.5 cm^2) using 20% Fe_2O_3 G_{62}-H_{170} and 20% Fe_2O_3 G_{62}-H_{170} -4% GO nanocomposite worm gels. Left column: images of QR codes after printing at room temperature. Right column: images of QR codes after being held at 2 °C for 15 min.

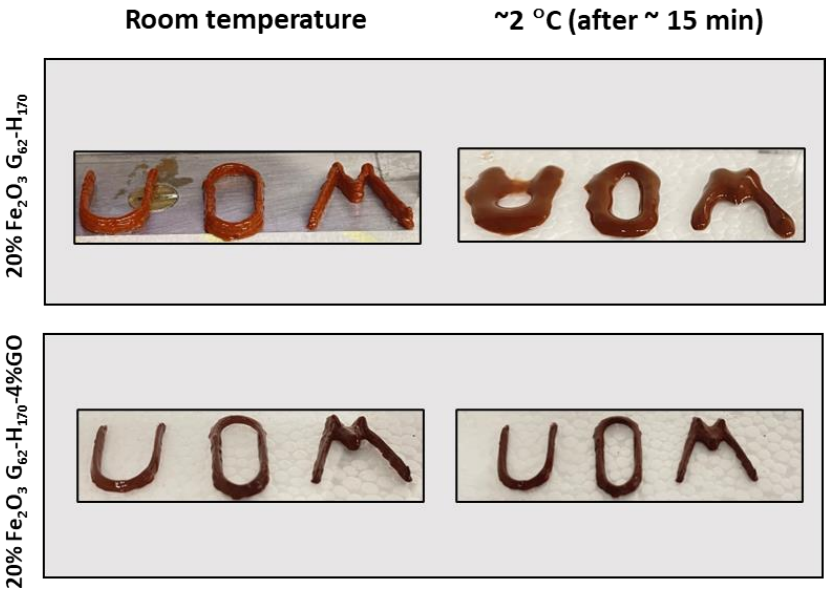

Figure S15. 3D-printed letters "UOM" of 20% Fe_2O_3 G_{62}-H_{170} and 20% Fe_2O_3 G_{62}-H_{170}-4% GO copolymer nanocomposite worm gels (left) at room temperature and (right) after cooling at ~2 °C for 15 min.

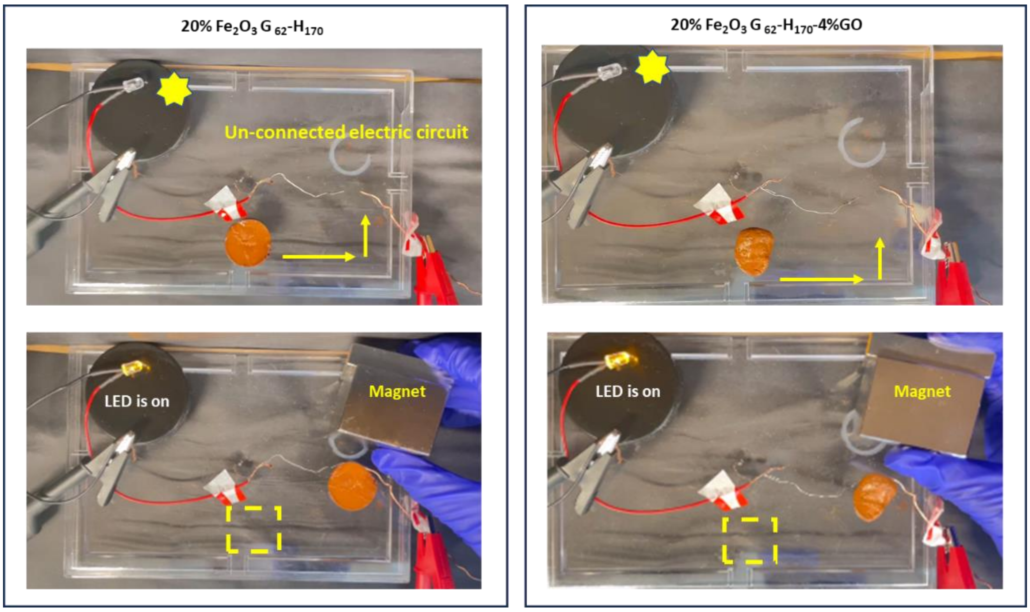

Figure S16. Digital photographs showing magnetically driven motion of 20% Fe_2O_3 G_{62}-H_{170} and 20% Fe_2O_3 G_{62}-H_{170}-4% GO gels. The magnet was used to move the gels without making physical contact, initially to the left and then forwards, to connect the electrical circuit and thus act as on/off switch. This demonstration was conducted at room temperature.

<u>**References**</u>

(1) Semsarilar, M.; Ladmiral, V.; Blanazs, A.; Armes, S. Anionic polyelectrolyte-stabilized nanoparticles via RAFT aqueous dispersion polymerization. *Langmuir* **2012**, *28* (1), 914-922.

(2) Wen, S.-P.; Saunders, J. G.; Fielding, L. A. Investigating the influence of solvent quality on RAFT-mediated PISA of sulfonate-functional diblock copolymer nanoparticles. *Polymer Chemistry* **2020**, *11* (20), 3416-3426.

(3) Warren, N. J.; Derry, M. J.; Mykhaylyk, O. O.; Lovett, J. R.; Ratcliffe, L. P.; Ladmiral, V.; Blanazs, A.; Fielding, L. A.; Armes, S. P. Critical dependence of molecular weight on thermoresponsive behavior of diblock copolymer worm gels in aqueous solution. *Macromolecules* **2018**, *51* (21), 8357-8371.

(4) Yue, Q.; Wen, S.-P.; Fielding, L. A. Preparation and characterisation of graphene oxide containing block copolymer worm gels. *Soft Matter* **2022**, *18* (12), 2422-2433.

(5) Williams, M.; Penfold, N.; Lovett, J.; Warren, N.; Douglas, C.; Doroshenko, N.; Verstraete, P.; Smets, J.; Armes, S. Bespoke cationic nano-objects via RAFT aqueous dispersion polymerisation. *Polymer Chemistry* **2016**, *7* (23), 3864-3873.

(6) d'Agosto, F.; Rieger, J.; Lansalot, M. RAFT‐mediated polymerization‐induced self‐assembly. *Angewandte Chemie International Edition* **2020**, *59* (22), 8368-8392.

(7) Blanazs, A.; Ryan, A.; Armes, S. Predictive phase diagrams for RAFT aqueous dispersion polymerization: effect of block copolymer composition, molecular weight, and copolymer concentration. *Macromolecules* **2012**, *45* (12), 5099-5107.

(8) Canning, S. L.; Smith, G. N.; Armes, S. P. A critical appraisal of RAFT-mediated polymerization-induced self-assembly. *Macromolecules* **2016**, *49* (6), 1985-2001.

(9) Warren, N. J.; Armes, S. P. Polymerization-induced self-assembly of block copolymer nano-objects via RAFT aqueous dispersion polymerization. *Journal of the American Chemical Society* **2014**, *136* (29), 10174-10185.

(10) Yue, Q.; Luo, Z.; Li, X.; Fielding, L. A. 3D printable, thermo-responsive, self-healing, graphene oxide containing self-assembled hydrogels formed from block copolymer wormlike micelles. *Soft Matter* **2023**.

(11) Fielding, L. A.; Lane, J. A.; Derry, M. J.; Mykhaylyk, O. O.; Armes, S. P. Thermo-responsive diblock copolymer worm gels in non-polar solvents. *Journal of the American Chemical Society* **2014**, *136* (15), 5790-5798.
